# Pluronic F-68 Improves Callus Proliferation of Recalcitrant Rice Cultivar *via* Enhanced Carbon and Nitrogen Metabolism and Nutrients Uptake

**DOI:** 10.3389/fpls.2021.667434

**Published:** 2021-06-02

**Authors:** Andrew De-Xian Kok, Nur Fatihah Mohd Yusoff, Rogayah Sekeli, Chien-Yeong Wee, Dhilia Udie Lamasudin, Janna Ong-Abdullah, Kok-Song Lai

**Affiliations:** ^1^Department of Cell and Molecular Biology, Faculty of Biotechnology and Biomolecular Sciences, Universiti Putra Malaysia, Serdang, Malaysia; ^2^Biotechnology and Nanotechnology Research Centre, Malaysian Agricultural Research and Development Institute (MARDI), Kuala Lumpur, Malaysia; ^3^Health Sciences Division, Abu Dhabi Women's College, Higher Colleges of Technology, Abu Dhabi, United Arab Emirates

**Keywords:** growth additive, growth promoting effects, mode of action, recalcitrant indica cv. MR 219, stress response

## Abstract

Pluronic F-68 (PF-68) is a non-ionic surfactant used in plant tissue culture as a growth additive. Despite its usage as a plant growth enhancer, the mechanism underlying the growth-promoting effects of PF-68 remains largely unknown. Hence, this study was undertaken to elucidate the growth-promoting mechanism of PF-68 using recalcitrant MR 219 callus as a model. Supplementation of 0.04% PF-68 (optimum concentration) was shown to enhance callus proliferation. The treated callus recorded enhanced sugar content, protein content, and glutamate synthase activity as exemplified in the comparative proteome analysis, showing protein abundance involved in carbohydrate metabolism (alpha amylase), protein biosynthesis (ribosomal proteins), and nitrogen metabolism (glutamate synthase), which are crucial to plant growth and development. Moreover, an increase in nutrients uptake was also noted with potassium topping the list, suggesting a vital role of K in governing plant growth. In contrast, 0.10% PF-68 (high concentration) induced stress response in the callus, revealing an increment in phenylalanine ammonia lyase activity, malondialdehyde content, and peroxidase activity, which were consistent with high abundance of phenylalanine ammonia lyase, peroxidase, and peroxiredoxin proteins detected and concomitant with a reduced level of esterase activity. The data highlighted that incorporation of PF-68 at optimum concentration improved callus proliferation of recalcitrant MR 219 through enhanced carbohydrate metabolism, nitrogen metabolism, and nutrient uptake. However, growth-promoting effects of PF-68 are concentration dependent.

## Introduction

Rice is one of the major staple foods serving more than half of the population of the world (Hadiarto and Tran, 2011). In Asian countries, rice provides nearly half of the total dietary carbohydrate and 50–80% of the daily calorie intake (Khush, 2005). Increasing rice production should be prioritized in order to sustain the ever-increasing world population (Sen et al., 2020). Through the advancement of modern biotechnology, genetic manipulation could serve as an alternative solution to meet the increasing demands of rice production (Low et al., 2018). However, genetic manipulation in *indica* rice cultivars remains a challenge. This is because *indica* rice cultivars share common recalcitrant properties toward *in vitro* regeneration responses. For instance, *indica* rice cultivars suffer from poor callus proliferation, low regeneration efficiency, a long regeneration period, and a low transformation rate (Sah et al., 2014). Therefore, in order to improve the *in vitro* responses of *indica* rice cultivar, optimization of the plant growth medium is required.

Additives are the key components in improving the *in vitro* responses of *indica* rice cultivars (Abiri et al., 2017; Kok et al., 2018). Some of the commonly used growth additives in plant tissue culture include Pluronic F-68 (PF-68), lignosulfonate, silver nitrate, silicon, coconut, and gibberellic acid (Biswas and Mandal, 2007; He et al., 2013; Yildirim and Turker, 2014; Irshad et al., 2018; Wan Abdullah et al., 2020). PF-68 is a non-ionic, copolymer surfactant that has been employed as an additive in both *in vitro* animal and plant cultures (Meier et al., 1999; Barbulescu et al., 2011). PF-68 has been widely utilized in animal cell suspension culture to protect and repair damaged cells from constant sparging and agitation (Meier et al., 1999). Further investigation of the application of PF-68 in yeast cells revealed that PF-68 is capable of interacting with the cell surface by increasing the permeability of the cell membrane through the formation of short-lived, transmembrane pores (King et al., 1991; Cho et al., 2007). Through enhanced membrane permeability, the nutrient uptake and cell growth were stimulated in cell culture (Shelat et al., 2013). However, the outcome of this interaction is concentration-dependent. At high concentration, PF-68 is able to disrupt the normal architecture of the lipid bilayer, resulting in a cell lysis (Cho et al., 2007).

Similar to animal cell culture, the application of PF-68 was found to enhance nutrient uptake and plant growth in plant tissue culture. In addition to that, application of PF-68 was demonstrated to enhance the release of anthraquinone into the medium in suspension cultures of *Morindacitrifolia*, suggesting an increase in membrane or cell wall permeability of *Morindacitrifolia* (Bassetti et al., 1995). A similar study was demonstrated, whereby secretion of a human granulocyte-macrophage colony-stimulating factor was stimulated when PF-68 was added into the cell suspension culture of transgenic *Nicotiana tabacum* (Cho et al., 2007). Besides, the application of PF-68 was reported to enhance shoot regeneration in *Citrus sinensis* (Curtis and Mirkov, 2012), *Pyrus communis* (Dashti et al., 2012), *Ricinus communis* (Kulathuran and Narayanasamy, 2015), and *Abelmoschus esculentus* (Irshad et al., 2018). Notably, PF-68 was demonstrated to improve the growth of roots, callus, and protoplast of *Solanum dulcamara* (Kumar et al., 1992), shoot regeneration of recalcitrant *Brassica napus* embryos (Barbulescu et al., 2011), and callus proliferation of recalcitrant *indica* rice (Kok et al., 2020). This implies that PF-68 could be a good candidate for plant cell growth and regeneration improvement of the recalcitrant cultivars.

An earlier study made by Kok et al. (2020) on PF-68 application has shown successful enhancement of a callus proliferation rate and a number of callus with root-like structure of MR 219 *indica* rice cultivar. Unfortunately, the underlying growth promoting mechanisms of PF-68 remains largely unknown. Hence, to close this gap and to maximize the usage of PF-68 as a growth additive, understanding its mode of action is crucial. Therefore, the present study aimed to investigate the mode of action of PF-68 in the growth enhancement of MR 219 *indica* rice cultivar.

## Materials and Methods

### Plant Materials

MR 219 cultivar was selected as a model of recalcitrant rice cultivar in this study. The seeds of recalcitrant Malaysian cultivar MR 219 used in this study were obtained from the Malaysian Agricultural Research and Development Institute (MARDI), Seberang Prai, Penang, Malaysia.

### Pluronic F-68

The analytical grade of PF-68 (10%) used in this research was purchased from Thermo Fisher Scientific, United States.

### Seed Sterilization and Growth Conditions

Surface sterilization of the seeds was performed according to previously described protocol by Lim and Lai (2017) with slight modifications. Firstly, mature seeds were de-husked and surface-sterilized using 70% (v/v) ethanol for 1 min, followed by 50% (v/v) Clorox, containing 6% sodium hypochlorite for 30 min. Subsequently, the seeds were washed with distilled water to remove remaining residues and allowed to be air-dried on a sterilized filter paper. The sterilized seeds were then transferred onto previously established a callus induction medium, containing a Gamborg's B5 basal medium (Gamborg et al., 1968), supplemented with 10-g/L maltose, 0.1-g/L L-glutamine, 0.1-g/L L-asparagine, 0.1-g/L L-arginine, 10-mg/L 1-naphthaleneacetic acid, and 1-mg/L 2,4-dichlorophenoxyacetic acid, pH 5.8 for 2-week incubation in the dark at 25 ± 2°C (Low et al., 2019). Subsequently, the calluses induced from the seed were transferred onto a Murashige and Skoog (MS) medium (Murashige and Skoog, 1962), containing 30-g/L sucrose without plant growth regulators (MSO), supplemented with PF-68 (0.04 and 0.10%; w/v) for 2-week incubation in the dark at 25 ± 2°C. Callus cultured onto an MS medium without PF-68 supplementation was used as the experimental control. Morphological changes in the callus were recorded at the end of the incubation period. The fresh weight (FW) of the callus was measured, and its constant dry weight (DW) was recorded after drying at 50°C in an oven for 5 days. The measurement was performed in triplicates with three biological replicates.

### Total Soluble Sugar Content

Total soluble sugar was measured using a phenol-sulfuric acid method (Terzi et al., 2014). Approximately, 0.25 g of calli were grounded into powder using liquid nitrogen. Subsequently, the powdered calli were homogenized in 3 ml of 80% (v/v) ethanol and centrifuged at 880 × *g* for 20 min. The pellet was discarded, and the supernatant was mixed with 1-ml 5% (v/v) phenol and 5-ml concentrated sulfuric acid per 1 ml of supernatant. The absorbance of the samples was measured at 565 nm by using a spectrophotometer (Implen GmbH, Germany). The corresponding concentration was determined, using glucose solution as a standard.

### Total Protein Content

The protein content of the callus was determined using the Bradford assay (Kruger, 1994). Approximately, 0.1-g calli were grounded into powder in liquid nitrogen and mixed with 900 μl of 50 mM of ammonium bicarbonate and 100 μl of 50-mM phenylmethylsulfonyl fluoride (PMSF). Subsequently, the mixture was vortexed, sonicated, and centrifuged according to Yang et al. (2019). Acetone precipitation was carried out according to Jiang et al. (2004). The protein pellet was dissolved at a ratio of 9:1 of 50-mM ammonium bicarbonate to 50-mM PMSF. The protein content in the extract was determined at 595 nm (Kruger, 1994). The corresponding concentration was determined, using bovine serum albumin as a standard.

### Glutamate Synthase Activity

Glutamate synthase (GOGAT) activity was measured, following a method described by Ertani et al. (2011). The following steps were performed at 4°C. For extraction of GOGAT activity, ~0.2 g of the samples was grounded into powder with the presence of liquid nitrogen. The powdered calli were then homogenized with 2 ml of extraction solution, containing 100-mM Hepes-NaOH at pH 7.5, 5-mM MgCl_2_, and 1-mM dithiothreitol. The extract was filtered through two layers of muslin cloth. After centrifugation at 20,000 × *g* at 4°C for 15 min, 100 μl of the supernatant was homogenized with 1 ml of mixture solution, containing 25-mM Hepes-NaOH at pH 7.5, 2-mM L-glutamine, 1-mM α-ketoglutaric acid, 0.1-mM NADH, and 1-mM Na_2_EDTA. The GOGAT activity was measured spectrophotometrically by monitoring NADH oxidation at 340 nm. The enzyme activity was expressed in μmol^−1^g^−1^FW, representing the amount of enzyme, catalyzing the oxidation of 1 μmol of NADH min^−1^.

### Phenylpropanoid Ammonia Lyase Activity

Phenylpropanoid ammonia lyase (PAL) activity was measured, following a protocol as described by Wang et al. (2016) with minor modifications. Approximately, 0.2 g of the sample calli was grounded into powder form in liquid nitrogen. Then, the powdered calli were mixed with 2 ml of the extraction buffer, comprising of 50-mM Tris-HCl buffer at pH 8.5, 5-mM Na_2_EDTA, 15-mM ß-mercaptoethanol, 1-mM PMSF, and 0.15% (w/v) polyvinylpyrrolidine. The extract was centrifuged at 12,000 × *g* at 4°C for 20 min. Twenty microliters of the extract were used to measure the proteins content, using the Bradford assay while the remaining extract was used to determine the PAL activity. A total of 500 μl of the extract was mixed with 3 ml of the reaction buffer solution, consisted of 50-mM Tris-HCl buffer (pH 8.5) and 12-mM L-phenyalanine. The mixture was then incubated at 30°C for an hour. The PAL activity was measured at 290 nm. The PAL activity was calculated based on a PAL standard curve made by cinnamic acid where one unit of PAL deaminates L-phenylalanine to trans-cinnamic acid. Specific activity of PAL was calculated by PAL activity at 290 nm (U), divided by PAL protein concentration (μg) and expressed as U/μg of protein.

### Malondialdehyde Content

Lipid peroxidation levels from the samples were determined through the malondialdehyde (MDA) assay. MDA is a lipid peroxidation product, which can be quantified in a reaction with thiobarbituric acid (Heath and Packer, 1968). Approximately, 0.2 g of the samples were grounded into powder, using liquid nitrogen and then homogenized with 2 ml of ice-cold phosphate buffered saline (PBS). Subsequently, the mixture was centrifuged at 12,000 × *g* for 15 min at 4°C. Two hundred microliters of the supernatant obtained were mixed with 800 μl PBS, 25 μl butylhydroxytoluene (8.8 mg/ml), and 500 μl of 50% (w/v) trichloroacetic acid. The mixture was left for 2 h on ice. Then, the mixtures were centrifuged at 12,000 × *g* for 15 min at 25°C. One milliliter of the sample was then mixed with 75 μl of 100 mM EDTA and 250 μl of 50-mM thiobarbituric acid. The mixture was boiled for 15 min and left to cool to room temperature. The MDA content in the sample was determined at 532 and 600 nm. The corresponding concentration was determined using tetramethoxypropane as a standard.

### Peroxidase Activity

Peroxidase activity was determined as described by Agostini et al. (1997) with modifications. Approximately, 2 g of samples were grounded into powder form, using liquid nitrogen and then homogenized with 0.8 ml of an extraction buffer, containing a 10-mM sodium acetate/acetic acid buffer at pH 4.0 and 1-M sodium chloride. After centrifugation at 5,000 × *g* for 5 min at 4°C, the extract was used to measure protein content, using the Bradford assay while the remaining extract was used to determine the peroxidase activity. A total of 2 μl of the extract was mixed with 1 ml of reaction mixture, containing 630-μM o-dianisidine, 500-μM H_2_O_2_, and a 100-mM sodium acetate/acetic acid buffer at pH 5.3. The peroxidase activity was determined immediately by measuring the absorbance at 470 nm. The peroxidase activity was measured based on the amount of an enzyme, forming 1 μmol of product in a minute, produced by o-dianisidine oxidation.

### Esterase Activity

Esterase activity was determined through a simple fluorometric method as described by Watanabe and Lam (2008). Fluorescein diacetate (FDA) was used as a fluorescent indicator of cell viability *via* endogenous esterase activity. Approximate, 0.2-g samples were grounded with liquid nitrogen. Then, the powdered calli were stained with a reaction mixture, containing 2.5 μg/ml of FDA in PBS. The mixture was left to incubate for 10 min at 25°C. Subsequently, the mixture was washed three times, using the reaction mixture, containing 2.5 μg/ml of FDA in PBS. For quantification of endogenous esterase activity, the resulting supernatants were used as samples for direct measurement of esterase activity *in vitro* by using a fluorescence microplate reader (TECAN infinite 200, Switzerland). The fluorescence value of the suspension was detected with an excitation wavelength at 440 nm and an emission wavelength at 550 nm.

### Callus Nutrient Ions Content Analysis via Atomic Absorption Spectrometry (AAS) Analysis

In nutrient ions content study, the callus specimen was dried in an oven at 70°C for 5 days. The specimen was processed as described previously by Karpiuk et al. (2016) with slight modifications. A dried specimen was homogenized into fine powder and kept in a desiccator until subsequent analysis. A mass of 1-g powdered specimen was weighed and transferred into a silica crucible and burnt in a muffle furnace. The muffle furnace temperature was gradually increased from room temperature to 530–550°C for 5 h. The cooled residue was then dissolved in 6 ml of 6-M HCl for an hour. The resulting solutions were then filtered through Whatman filter papers into a volumetric flask. The filtered solution was then transferred to the AAS instrument (Thermo Fisher Scientific, United States) to measure the nutrients content by comparison with the standards. The operation conditions used to operate the AAS instrument were as recommended by the manufacturer.

### Proteomic Analysis

In proteomic analysis, plant samples were grounded into fine powder, using liquid nitrogen and homogenized at a ratio of 9:1, consisting of 50 mM of ammonium bicarbonate to 50-mM PMSF. The mixture was then vortexed, sonicated, and centrifuged according to Yang et al. (2019), and solubilized proteins were collected. Desalting was carried out, using an acetone precipitation method (Jiang et al., 2004). The protein pellet was dissolved at a ratio of 9:1 (50-mM ammonium bicarbonate to 50-mM PMSF). The protein content in the extract was determined at 595 nm through the Bradford assay (Kruger, 1994). The protein sample was added with dithiothreitol to a final concentration of 10 mM and then incubated on an orbitor shaker at 25 ± 2°C for 30 min. Then, iodoacetamide was added into the mixture to a final concentration of 10 mM, and the mixture was further incubated in darkness at room temperature for 30 min. Trypsin was added into the protein mixture at a ratio of 1:20 for trypsin to protein (w/w) and incubated overnight at room temperature. The sample was dried in a SpeedVac vacuum concentrator (Thermo Fisher Scientific, United States). Following which, the dried sample was dissolved in 200 μl of molecular biology grade water and dried in the Speedvac. This step was repeated twice. The sample was then stored at −80°C until further analysis.

Nano liquid chromatography tandem-mass spectrometry (nano LC-MS/MS) was performed, using the Dionex 3,000 Ultimate RSLCnano (Thermo Fisher Scientific, United States). An aliquot of a 2-μl-digested-proteins sample was injected into the EASY-Spray Column Acclaim PepMapTM C18 100 (A0, 2 μm particle size, 50 μm id × 15 cm) at 35°C. The sample elution process was performed similarly as described by Yang et al. (2019). The eluent from the LC was directly introduced into the mass spectrometer (Orbitrap Fusion – Thermo Fisher Scientific, United States). The instrument was operated in the data-dependent mode. Full-scan spectra were collected Orbitrap MS (OTMS1) using parameters defined by a previous study (Yang et al., 2019). Only precursors with an assigned monoisotopic m/z and a charge state of 2 to 7 were further analyzed for MS2. All precursors were filtered using a 20-s dynamic exclusion window and an intensity threshold of 5,000. The precursors were fragmented by collision-induced dissociation (CID) and higher-energy collisional dissociation (HCD) at a normalized collision energy of 30 and 28%. The data were analyzed, using the Thermo Scientific^TM^ Proteome Discoverer^TM^ Software 2.1.

### Real-Time PCR Analysis

Total RNA was isolated from the powdered calli, incubated in three treatments (control, 0.04%, 0.10% PF-68) using RNeasy Plant Mini Kit (Qiagen, Germany), following the protocol described in Lai and Masatsugu (2013). First-strand cDNA was synthesized from 1 μg of isolated total RNA using QuantiNova Reverse Transcription Kit (Qiagen, Germany). The primers were designed (Supplementary Table 1), using Primer-Blast from the National Center for Biotechnology Information (NCBI) and synthesized by Integrated DNA Technologies (IDT, United States). Real-time PCR was performed, using a aBio-Rad CFX96 system (Bio-Rad, United States) with QuantiNova SYBR Green PCR (Qiagen, Germany), following a protocol described in Lai et al. (2011). The PCR reaction conditions used were as follows: 95°C for 30 s, followed by 40 cycles of 95°C for 5 s and 60°C for 5 s. Three biological replicates and three technical replicates were performed for each sample. The data were analyzed, using the Bio-Rad CFX Manager 3.1 software. The relative expression levels (2^−Δ*ΔCT*^) were calculated according to Livak's method (Livak and Schmittgen, 2001). The reference genes used in this study were rice *cyclophilin* (*OsCYC*) and *ubiquitin 5* (*OsUBQ5*).

### Statistical Analysis

All data presented were the average ± standard error mean (SEM) of three biological replicates with three technical replicates. The data were analyzed, using one-way analysis of variance at the significant level of *p* < 0.05 between each treatment, using the Statistical Package for the Social Sciences version 20 (IBM Corp., Armonk, United States).

## Results

### Growth-Promoting Response of PF-68 Without the Presence of Plant Hormone

**Figure 1:**
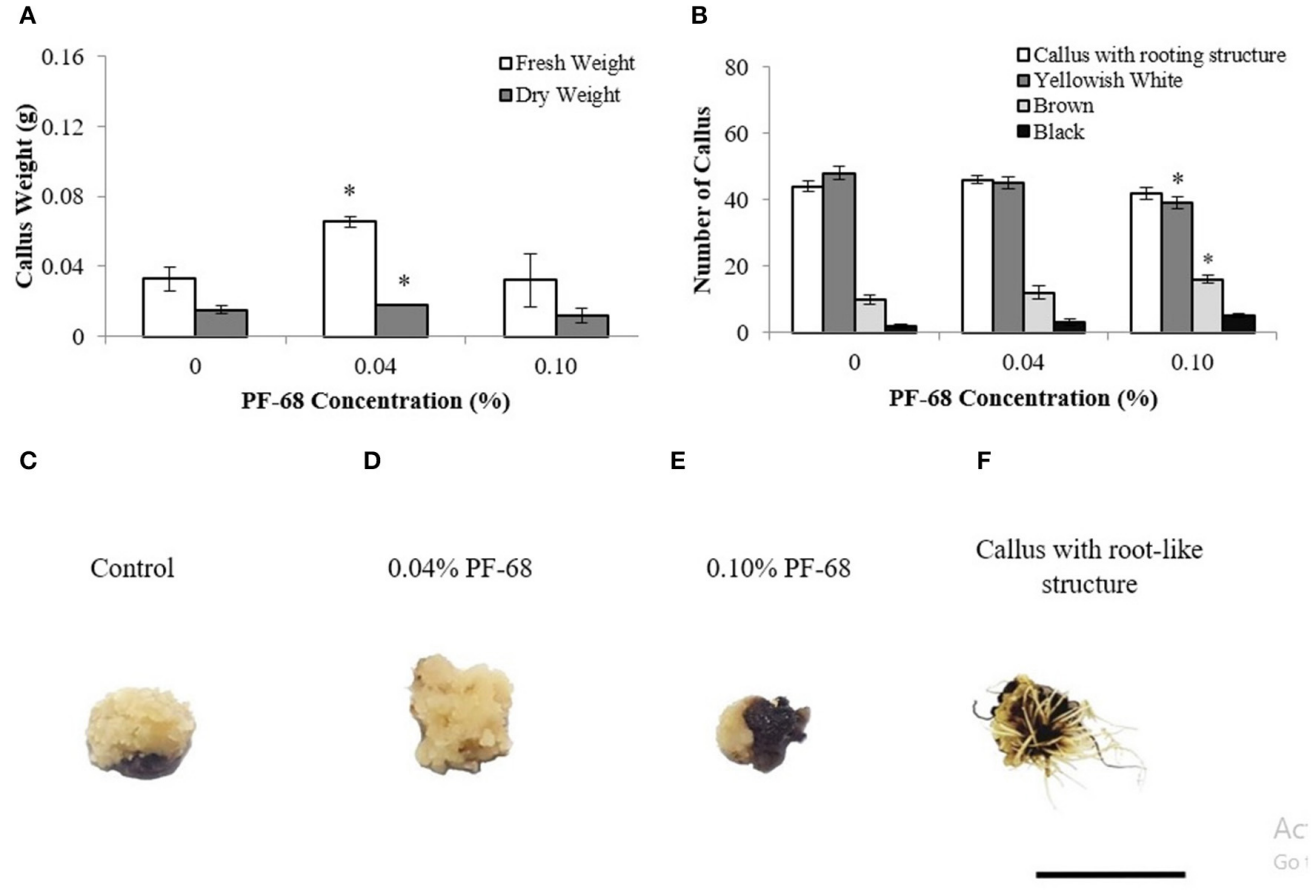
Data obtained from calli proliferated on Murashige and Skoog (MS) medium supplemented with different PF-68 concentrations without plant hormones (MSO) for 3 weeks. **(A)** Mean fresh and dry weights recorded on 3 weeks old calli; **(B)** callus morphology on MSO (control), MSO +0.04% and MSO +0.10% PF-68; **(C)** control callus at week 3; **(D)** callus on MSO +0.04% PF-68 at week 3; **(E)** callus on MSO +0.10% PF-68 at week 3; **(F)** callus with root-like structure at week 3. Data shows the mean of three biological replicates. Asterisks (*) indicate statistical significant difference at *p* < 0.05 in Dunnet's test. Scale bars represent 0.5 cm. Error bars represent standard error mean.

An earlier study on PF-68 in callus proliferation revealed that optimum concentration of PF-68 (0.04%) significantly enhanced callus proliferation of MR 219 (Kok et al., 2020). Meanwhile, high concentration of PF-68 (0.10%) was demonstrated to induce stress response (Kok et al., 2020). In this study, plant hormones were removed from the callus proliferation medium in order to investigate the mechanism governing the growth-promoting response of PF-68 as a plant additive without the interference of plant hormones. Based on the results shown in Figure 1A, the application of 0.04% PF-68 significantly enhanced MR 219 callus proliferation in the MSO medium by 68.1% and 15.02% in FW and DW, respectively.

Most of the calli (Figure 1B) cultured on both control and PF-68 were compact, dry, and yellowish-white (Figures 1C,D). As the concentration of PF-68 increased, decreasing numbers of yellowish-white calli were observed (Figure 1B). The highest number of yellowish-white calli (80%) was recorded in the MSO (control) compared with 75% with 0.04% PF-68 supplementation and 65.00% with 0.10% PF-68 supplementation (Figure 1B). Increasing numbers of brown (Figure 1E) and black (Figure 1F) calli were also noted. The highest number of brown and black calli recorded in 0.10% PF-68 was 26.67 and 8.33%, respectively (Figure 1B). Besides, the application of PF-68 enhanced the numbers of callus with root-like structure (Figure 1F). Supplementation with 0.04% PF-68 recorded the highest number at 76.67% (Figure 1B).

### Biochemical Assessments of PF-68 at Optimum and High Concentrations

**Figure 2:**
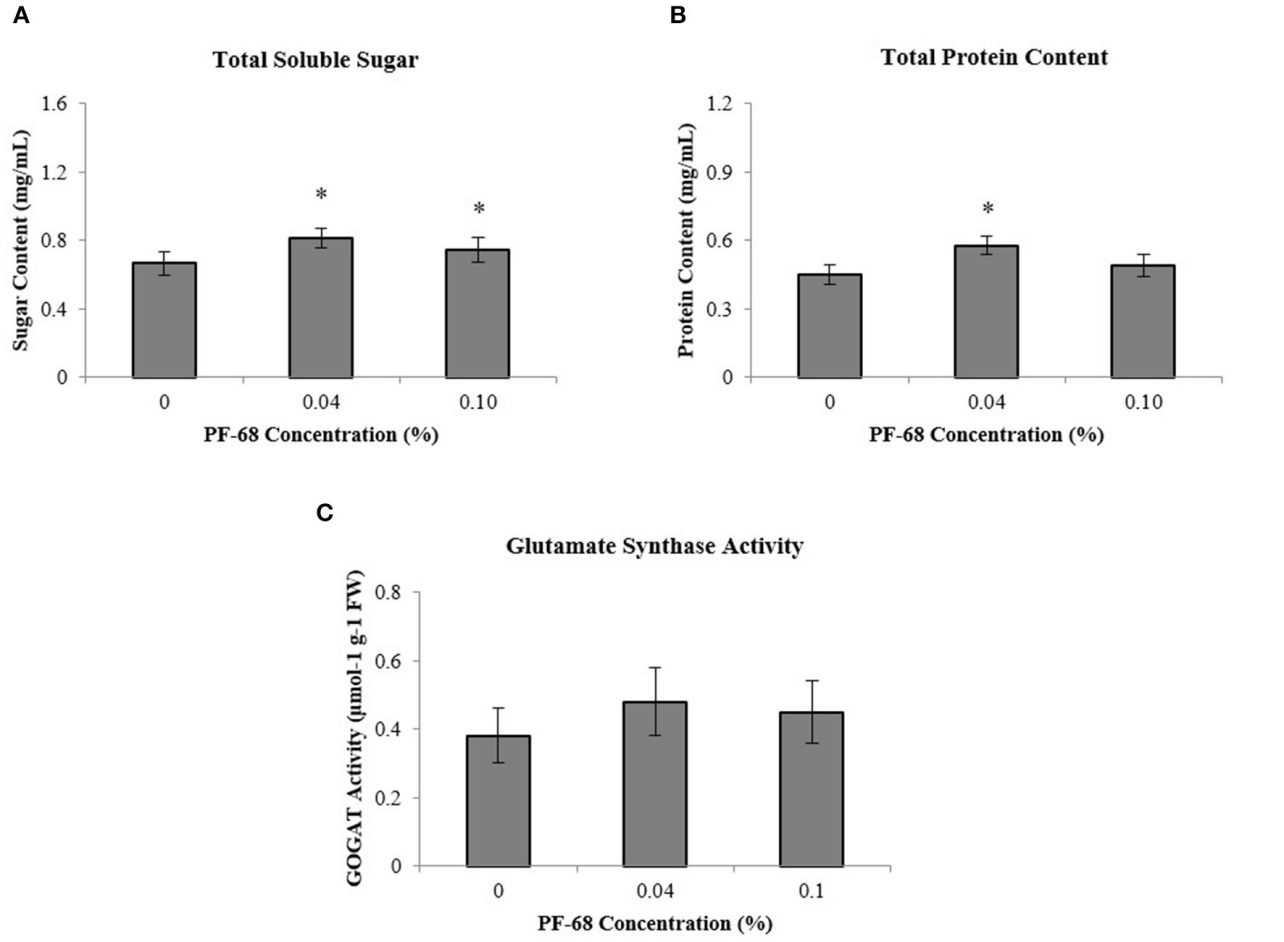
Biochemicals analysis was performed on extracts of calli grown on Murashige and Skoog (MS) medium supplemented with different PF-68 concentrations without plant hormones (MSO). Control (MSO), optimum (MSO +0.04% PF-68), and high (MSO +0.10% PF-68). **(A)** Total soluble sugar; **(B)** total protein content; **(C)** glutamate synthase (GOGAT) activity. Data shows the mean of three biological replicates. Asterisks (*) indicate values were significantly different from those of the control callus at *p* < 0.05. Error bars represent SD of three biological replicates.

To further investigate the underlying growth-promoting mechanism of PF-68 in callus proliferation, biochemical assessments were performed on MSO, 0.04% PF-68 and 0.10% PF-68. A significant increment of total sugar content was found in both calli grown on 0.04% PF-68 (0.81 mg/ml) and 0.10% PF-68 (0.75 mg/ml), as compared with the control (0.67 mg/ml) (Figure 2A). Similarly, a significant increase of proteins content was recorded in calli grown on 0.04% PF-68 (0.58 mg/ml) and 0.10% PF-68 (0.49 mg/ml), compared with the control (0.45 mg/ml) (Figure 2B). The increases in proteins content were generally consistent with the increases in GOGAT activity as recorded in calli grown on 0.04% PF-68 with 0.48-μmol/g protein compared with the control 0.38-μmol/g protein (Figure 2C).

**Figure 3:**
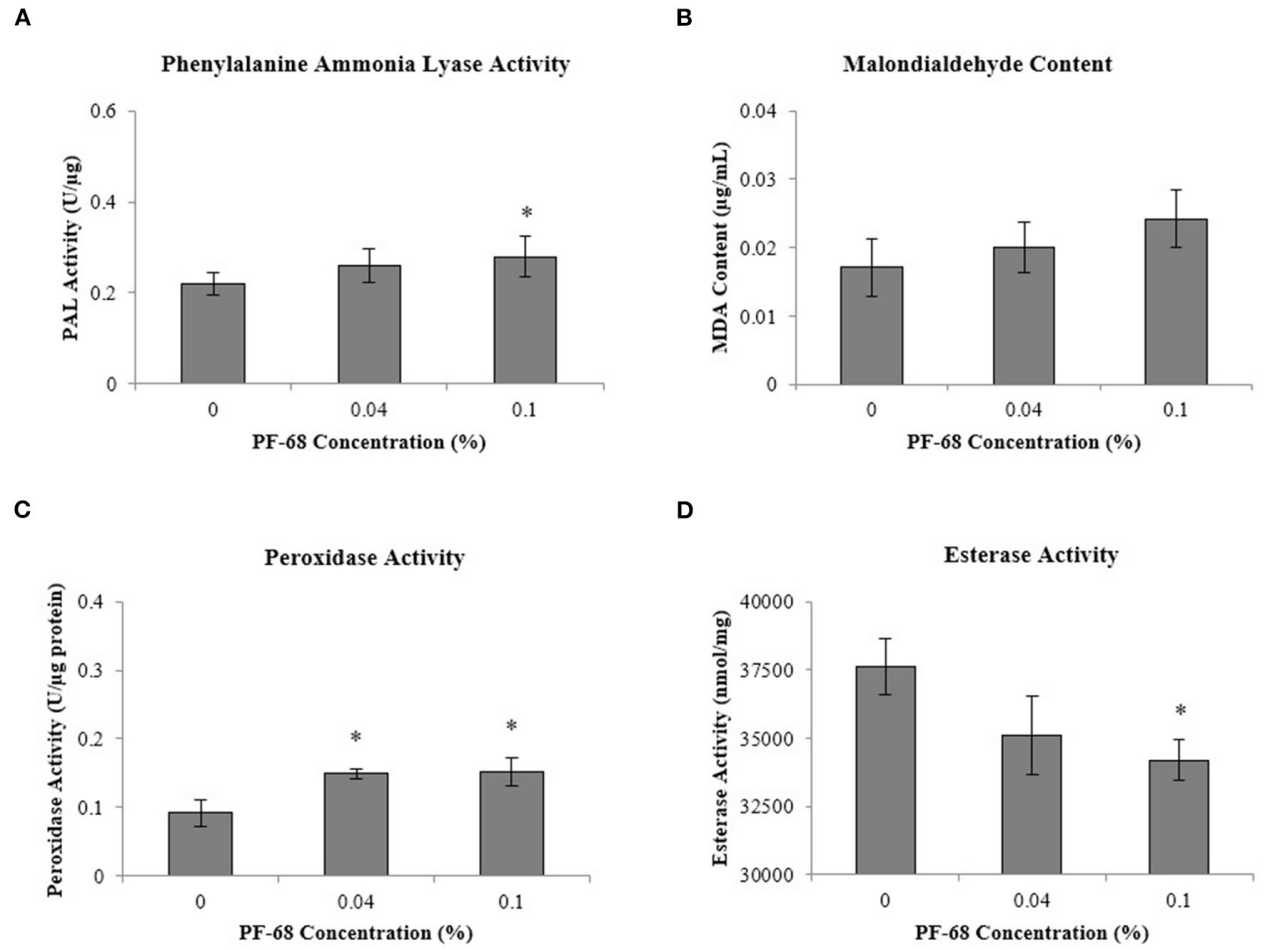
Biochemical analysis performed on extracts of calli grown on Murashige and Skoog (MS) medium supplemented with different PF-68 concentrations without plant hormones (MSO). Control (MSO), optimum (MSO +0.04% PF-68), and high (MSO +0.10% PF-68). **(A)** Phenylalanine lyase (PAL) activity; **(B)** malondialdehyde (MDA) content; **(C)** peroxidase activity; **(D)** esterase activity. Data shows the mean of three biological replicates. Asterisks (*) indicate values were significantly different from those of the control callus at *p* < 0.05. Error bars represent SD of three biological replicates.

Besides, stress-related biochemical assessments performed in order to investigate the underlying mechanism induced by PF-68 at high concentration have demonstrated that the highest PAL activity was detected in calli grown on 0.10% PF-68 (0.28-U/μg protein) (Figure 3A), which corroborated with its highest MDA content of 0.024-U/μg protein (Figure 3B) and highest peroxidase activity of 0.15-U/μg protein (Figure 3C). In determination of esterase activity, calli grown on 0.10% PF-68 were recorded to have the lowest esterase activity (34,204.50-nmol/ng protein), as compared with 0.04% PF-68 (35,091.67-nmol/ng protein) and control (37,620.17-nmol/ng protein) (Figure 3D).

### Nutrient Ions Content Analysis on MR 219 Callus Treated With PF-68

Nutrients availability is crucial to plant growth and development. In order to evaluate the effects of PF-68 on nutrients uptake in MR 219 callus, nutrient ion analysis *via* atomic absorption spectrometry was performed. Table 1 reveals that calli grown on 0.04% PF-68 contained higher amounts of macronutrients (K, Mg, and Ca) and micronutrients (Fe, Zn, Cu, and Mn), except for Na, which had the same amount as compared with the control. However, calli grown on 0.10% PF-68 contained higher amounts of Ca, Fe, Zn, Cu, and Mn compared with the control. Among these macro- and micronutrients tested, K had the highest increment detected in 0.04% PF-68 (42,600 ppm) when compared with the control (40,600 ppm).

**Table 1:** The concentration of nutrient ions content in control callus, and calli supplemented with 0.04% PF-68 and 0.10% PF-68 after 4 weeks of incubation.

**Nutrient content (ppm)**								
**Samples**	**K**	**Mg**	**Ca**	**Na**	**Fe**	**Zn**	**Cu**	**Mn**
Control	40,600	1,500	2,200	700	270.00	85.89	2.08	101.00
0.04% PF-68	42,600	1,600	2,500	700	281.40	89.84	2.18	125.20
0.10% PF-68	39,500	1,500	2,400	600	291.50	87.08	2.17	127.10

### Comparative Proteomic Analysis on MR 219 Callus Treated With PF-68

Comparative proteomic analysis was carried out between the control, optimum (0.04%), and high concentrations (0.10%) of PF-68 in order to shed light on possible roles of PF-68 in callus growth. Differentially expressed proteins were identified in three different comparing groups; namely, between control and optimum concentration, control and high concentration, and optimum and high concentration. Pearson correlation values in these three groups were of high confidence, indicating the samples used between the different treatment groups were linearly related (Supplementary Figure 1). In addition, principal component analysis revealed good separation between the treatment groups, indicating significant changes in the proteomic abundance between each group (Supplementary Figure 1).

**Figure 4:**
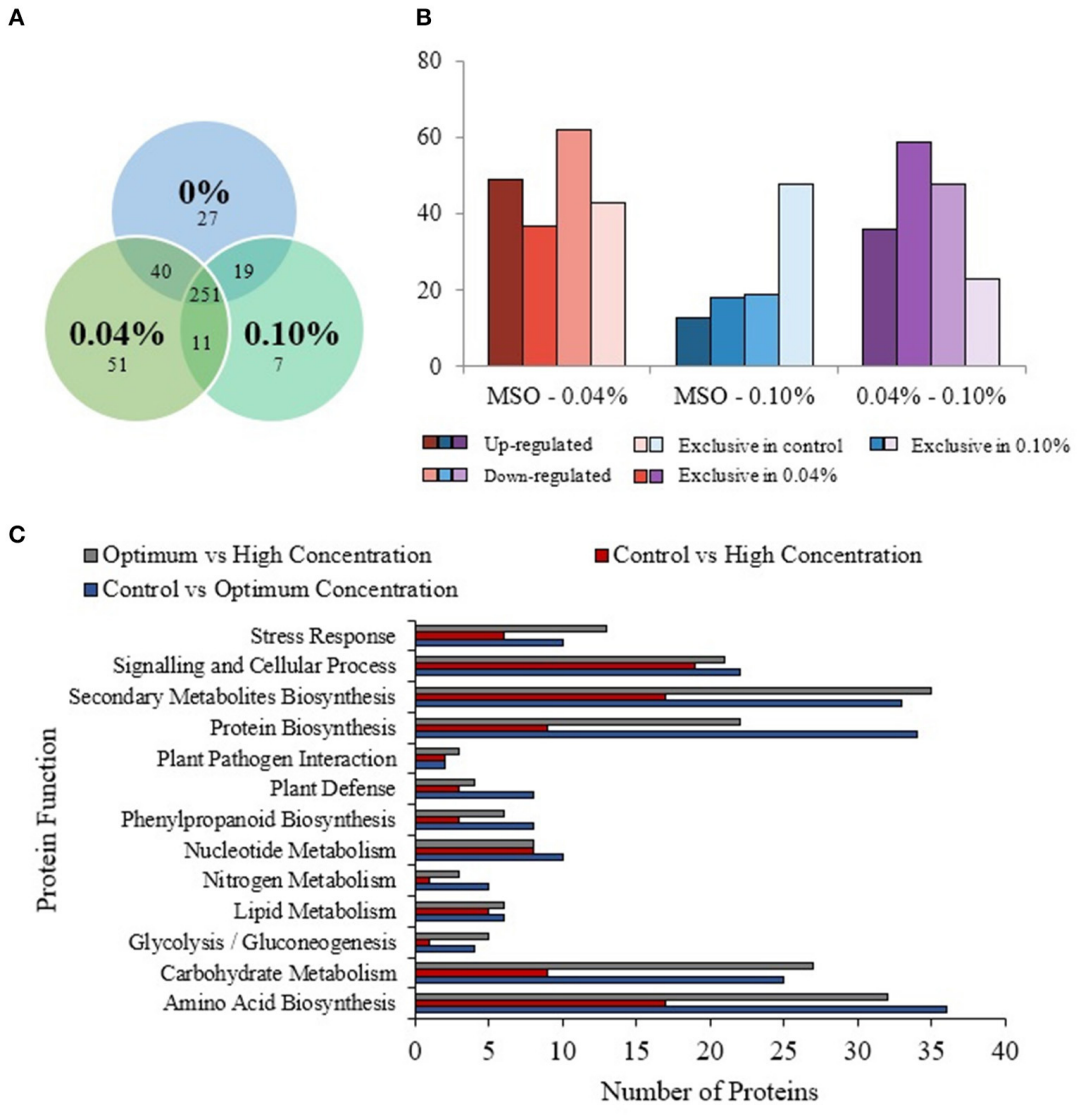
Comparative proteomic analysis of extracts from calli exposed to different PF-68 concentrations. Control (0% PF-68), optimum (0.04% PF-68), and high (0.10% PF-68). **(A)** Venn diagram of the total proteins obtained from the comparison between three treatments. **(B)** Total differentially expressed proteins identified in three treatment groups. **(C)** KEGG-pathway analysis of differentially expressed proteins identified in three treatment groups.

A total of 337 proteins from the control, 353 proteins from the optimum concentration, and 288 proteins from high concentration were successfully identified (Figure 4A). A total of 251 similar proteins were shared between the three treatment groups. In comparison between the control and optimum concentrations, a total of 380 proteins were identified in these two treatments. A total of 315 proteins were identified in both control and high concentrations. When compared between optimum and high concentrations, a total of 320 proteins were identified in both treatments. Based on the analysis, 49 proteins were upregulated, and 62 proteins were downregulated in optimum concentration as compared with the control (Figure 4B; Supplementary Figure 1 and Supplementary Table 2). In comparison between the control and high concentrations, 13 proteins were found to be upregulated, while 19 proteins were downregulated (Figure 4B; Supplementary Figure 1 and Supplementary Table 3). A total of 36 proteins were upregulated, and 48 proteins were downregulated in optimum as compared with a high concentration of PF-68 (Figure 4B; Supplementary Figure 1 and Supplementary Table 4). In addition, 37 proteins were exclusive to the optimum concentration, and 43 proteins were exclusive to the control when compared between the control and optimum concentrations. A total of 18 proteins were exclusive to the high concentration, and 48 proteins were exclusive to the control when compared between the control and high concentrations. When compared between the optimum and high concentrations, 59 proteins were exclusive to the optimum concentration, and 23 proteins were exclusive to the high concentration (Figure 4B). The differentially expressed proteins identified were subjected to Kyoto Encyclopedia of Genes and Genomes (KEGG) pathway analysis, which revealed that PF-68 supplementation affected proteins that are involved in carbohydrate metabolism, amino acid biosynthesis, protein biosynthesis, and secondary metabolites biosynthesis (Figure 4C). Besides, gene expression of selected proteins was observed to be in a similar trend with the proteome profile (Supplementary Figure 2).

## Discussion

The recalcitrant property of *indica* rice cultivars has been one of the major obstacles in *in vitro* regeneration of MR 219, particularly poor callus induction and proliferation. Various attempts were made in order to improve *in vitro* regeneration of MR 219, such as the application of plant additives into the culture medium (Low et al., 2019). A recent study on the application of PF-68 has successfully improved a callus proliferation rate in MR 219 in the presence of plant hormones (Kok et al., 2020). Hence, the present study was undertaken in order to further elucidate the mechanisms governing the growth-promoting response of PF-68 during callus proliferation.

Application of optimum PF-68 concentration alone, without the supplementation of plant hormone, improved callus proliferation of MR 219 rice (Figure 1). These observations were consistent with the previous study made on the application of PF-68 with the presence of plant hormones (Kok et al., 2020). This suggests that PF-68 can act as a growth additive independent of plant growth hormones.

Application of PF-68 was found to be adsorbed onto the cell surfaces, providing protection against physical and chemical stresses in animal cell culture. The interaction between PF-68 and membrane permeability has been studied in yeast, animal cell culture, and plant cell culture (King et al., 1991; Meier et al., 1999; Cho et al., 2007). The incorporation of PF-68 at low concentration has been demonstrated to influence membrane permeability (King et al., 1991). Through enhanced membrane permeability, it was suggested that PF-68 was able to promote nutrient uptake and cell growth in animal cell culture (Shelat et al., 2013).

Soluble sugar molecules such as sucrose, glucose, and fructose play an important role in sugar sensing and plant development. High-sugar content can promote cell growth and carbohydrate storage by producing carbon and energy required for plant growth and development (Eveland and Jackson, 2012). Starch, a branched glucose polymer, also functions as a reserve carbohydrate in plants (Tetlow and Emes, 2014). In the present study, accumulation of soluble sugar was observed in callus treated with optimum PF-68 concentration (Figure 2A). Similar findings were reported in the application of PF-68 during callus growth of *S. dulcamarain* (Kumar et al., 1992). In response to PF-68, the enhanced accumulation of soluble sugar was reflected with the increases in tissue weights of *S. dulcamarain* (Kumar et al., 1992). Besides, comparative proteomic analysis revealed that cytosolic invertase 1 protein was found to be exclusive in callus treated with optimum PF-68 (Supplementary Tables 2, 4). In plants, invertase plays a vital role in hydrolyzing sucrose into glucose and fructose, and providing carbon nutrient supply required for cellular biosynthesis and sugar signal transduction (Ruan et al., 2010). For instance, point mutation on *cytosolic invertase 1* gene in *Arabidopsis* was shown to cause sucrose accumulation and reduced plant growth (Qi et al., 2007). Another study reported that mutated *cytosolic invertase 1* gene exhibited delayed flowering and partial sterility in rice (Jia et al., 2008). Detection of invertase protein exclusively found in callus treated with optimum PF-68 concentration suggested that production of glucose and fructose from sucrose was enhanced.

Besides, an increase in abundance of alpha-amylase isozyme 3A was detected in callus treated with optimum PF-68 concentration (Table 2). During carbohydrate metabolism, alpha-amylase is produced in abundance, and it is an essential enzyme in plants for catalyzing the hydrolysis of alpha-1,4-glucosidic bonds in starch (Zeeman et al., 2010). In plants, starch degradation is often dependent on carbon availability. For instance, starch degradation was halted when carbon availability was high, while starch degradation was stimulated when low carbon availability was detected in plants (Weise et al., 2006). Supporting this, a 1,4-alpha-glucan-branching enzyme was found to be in low abundance in the callus treated with optimum PF-68 concentration (Supplementary Tables 2, 4). Starch branching enzyme (SBE) like 1,4-alpha-glucan-branching enzyme is one of the major enzymes involved in starch biosynthesis in plants. This SBE can influence the structure of starch in terms of frequency and branch chain length (Tetlow and Emes, 2014). Therefore, the decrease in SBE abundance, as recorded in callus treated with optimum PF68 concentration, suggests that the production of starch has greatly reduced. This further supports that the carbohydrate metabolism was enhanced in order to provide a steady supply of energy and carbon for plant growth and development (Zeeman et al., 2010).

**Table 2:** Top 20 proteins showing significant abundance difference (together with their accession numbers) between control and optimum PF-68 concentrations.

**No**	**Protein name**	**Uniprot accession no**.	**General function**	**Difference in protein abundance**
**Up-regulated proteins found in 0.04% PF-68 when compared to control (0% PF-68)**				
1	Alpha-amylase isozyme 3A	P27932	Carbohydrate Metabolism	3.33631
2	Heat shock 70 kDa protein BIP4	Q75HQ0	Stress Response	2.66305
3	60S ribosomal protein L10a	B7F845	Protein Biosynthesis	2.33374
4	Non-specific lipid-transfer protein 1	Q0IQK9	Transporter Protein	2.07060
5	Obg-like ATPase 1	Q6Z1J6	Stress Response	1.53549
6	GDP-mannose 3,5-epimerase 2	Q2R1V8	Secondary Metabolites Biosynthesis	1.49322
7	Cysteine proteinase inhibitor 4	Q5N806	Plant Defense	1.44843
8	Actin-2	A3C6D7	Signaling and Cellular Process	1.43792
9	Germin-like protein 3-6	Q851K1	Plant Defense	1.38362
10	Nucleosome assembly protein 1;1	Q5VND6	Translational Modification	1.31477
11	Cyanate hydratase	Q9FWK4	Nitrogen Metabolism	1.28015
12	40S ribosomal protein S21	P35687	Protein Biosynthesis	1.05259
13	Proteasome subunit alpha type-7-B	Q0J006	Protein Biosynthesis	1.02611
14	Elongation factor 1-delta 1	Q40680	Protein Biosynthesis	0.99770
15	Ferredoxin–NADP reductase, root isozyme, chloroplastic	P41345	Transporter Protein	0.98280
16	Histone H3.2	Q2RAD9	Signaling and Cellular Process	0.96914
17	Cinnamyl alcohol dehydrogenase 7	Q0JA75	Phenylpropanoid Biosynthesis	0.92934
18	Actin-7	P0C540	Signaling and Cellular Process	0.91432
19	Thioredoxin-like protein CXXS1	Q0J9V5	Translational Modification	0.86712
20	Delta-aminolevulinic acid dehydratase, chloroplastic	Q5Z8V9	Secondary Metabolites Biosynthesis	0.84856
**Down-regulated Proteins found in 0.04% PF-68 when compared to control (0% PF-68)**				
1	Villin-5	Q0J716	Signaling and Cellular Process	−1.31358
2	Elongation factor Ts, mitochondrial	Q6ZJS7	Protein Biosynthesis	−1.30464
3	Peroxidase 2	Q0D3N0	Phenylpropanoid Biosynthesis	−1.27717
4	Protein argonaute 1A	Q6EU14	N/A	−1.22130
5	Neutral ceramidase	Q0JL46	Lipid Metabolism	−1.09147
6	Peroxiredoxin-2C	Q9FR35	N/A	−1.01497
7	Glutamate dehydrogenase 2, mitochondrial	Q33E23	Amino Acid Biosynthesis	−0.97888
8	12-oxophytodienoate reductase 1	Q84QK0	Lipid Metabolism	−0.94814
9	NADP-dependent malic enzyme, chloroplastic	P43279	Carbohydrate Metabolism	−0.89326
10	ATP synthase subunit alpha, mitochondrial	P0C522	N/A	−0.88513
11	Putative 12-oxophytodienoate reductase 5	Q69TI0	Lipid Metabolism	−0.87959
12	Profilin LP04	Q5VMJ3	Signaling and Cellular Process	−0.84333
13	60S acidic ribosomal protein P0	P41095	Protein Biosynthesis	−0.80776
14	ATP synthase subunit beta, mitochondrial	Q01859	N/A	−0.79720
15	Actin-depolymerizing factor 2	Q9AY76	Signaling and Cellular Process	−0.78577
16	2-Cys peroxiredoxin BAS1, chloroplastic	Q6ER94	Plant Defense	−0.75732
17	Oryzain gamma chain	P25778	Protein Biosynthesis	−0.75668
18	Clathrin heavy chain 2	Q2QYW2	N/A	−0.73074
19	Ubiquitin-fold modifier 1	Q94DM8	Translational Modification	−0.72169
20	L-ascorbate peroxidase 1, cytosolic	Q10N21	Amino Acid Biosynthesis	−0.72137

*The list is sorted in descending order in accordance to decreased and then increased in protein abundance of each protein*.

*N/A, not available*.

Aside from soluble sugar accumulation, total protein accumulation plays an important role during plant growth and development as well. Proteins accumulation in plant is often dependent on the availability of nitrogen supply. Nitrogen is one of the essential building blocks of amino acids. In many natural environments, nitrogen is often one of the limiting nutrients, and the decrease in nitrogen availability is often the reason for reduced plant growth. In the present study, enhanced GOGAT activity and increased in abundance of NADH-GOGAT protein were detected in callus treated with optimum PF-68 concentration (Figure 2C and Supplementary Table 2). In plants, GOGAT is one of the key proteins involved in amino acid biosynthesis, specifically nitrogen metabolism. GOGAT isoenzymes catalyze the transfer of the amido nitrogen of glutamine to 2-oxoglutarate, using either pyridine nucleotides (NADH dependent) or ferredoxin (ferredoxin dependent) as a reductant (Konishi et al., 2014). Besides, Chichkova et al. (2001) previously reported that overexpression of the *NADH-GOGAT* gene in the transgenic tobacco plant was shown to enhance dry weight and total carbon and nitrogen contents. Enhanced GOGAT activity and increased in abundance of NADH-GOGAT protein were observed to be coherent accumulation of total proteins in the callus treated with optimum PF-68 concentration (Figure 2). This indicates that the application of 0.04% PF-68 may play an active role in nitrogen assimilation in callus. The increased nitrogen content will then be used as a source of building blocks for amino acids and subsequently enhances the biosynthesis of proteins, which are crucial to plant growth (Rafiq et al., 2010).

Further comparative proteome analysis revealed an increase in abundance of ribosomal proteins (60S ribosomal protein L10a and 40S ribosomal protein S21) found in callus treated with optimum PF-68 concentration when compared with both control and high PF-68 concentration (Table 3). Ribosomal proteins are well-known for their roles in mediating protein synthesis and maintaining the stability of the ribosomal complex. The ribosome complex, as a whole, ensures the process of initiation of protein synthesis, amino acid assembly, and termination to occur appropriately in the cells (Moin et al., 2016). Regulation of ribosomal proteins is developmental dependent. For instance, high levels of ribosomal proteins were found in plant tissues with active division activities (Ferreyra et al., 2010). Ito et al. (2000) reported that disruption of ribosomal proteins resulted in developmental defects in *Arabidopsis*, such as an aberrant leaf, retarded root growth, and late flowering. Hence, the increase in the abundance of ribosomal proteins detected in the callus treated with optimum PF-68 concentration may play an important role during cell growth and development. Besides, similar findings were reported on the application of PF-68 on *S. dulcamarain*, whereby enhanced protein accumulation was observed during callus growth (Kumar et al., 1992).

**Table 3:** Top 20 proteins showing significant abundance difference (together with their accession numbers) when compared between control and high PF-68 concentrations.

**No**	**Protein name**	**Uniprot accession no**.	**General function**	**Difference in protein abundance**
**Up-regulated Proteins found in 0.10% PF-68 when compared to control (0% PF-68)**				
1	Probable histone H2A.1	Q6ZL43	Signaling and Cellular Process	2.63844
2	Thioredoxin Y, chloroplastic	Q5JMR9	Translational Modification	1.12346
3	Ferredoxin-dependent glutamate synthase, chloroplastic	Q69RJ0	Amino Acid Biosynthesis	0.99525
4	Ferredoxin–NADP reductase, root isozyme, chloroplastic	P41345	N/A	0.97960
5	Probable L-ascorbate peroxidase 6, chloroplastic/mitochondrial	P0C0L1	Amino Acid Biosynthesis	0.95538
6	Glutamate synthase 1 [NADH], chloroplastic	Q0JKD0	Amino Acid Biosynthesis	0.93512
7	Chaperone protein ClpC1, chloroplastic	Q7F9I1	Translational Modification	0.88085
8	Cupincin	Q852L2	N/A	0.75954
9	Phenylalanine ammonia-lyase	P14717	Secondary Metabolites Biosynthesis	0.74090
10	Non-specific lipid-transfer protein 3	Q2QYL3	Plant Defense	0.66712
11	Putative 12-oxophytodienoate reductase 4	Q69TH8	Secondary Metabolites Biosynthesis	0.58307
12	Proteasome subunit alpha type-3	Q9LSU0	Protein Biosynthesis	0.54185
**Down-regulated Proteins found in 0.10% PF-68 when compared to control (0% PF-68)**				
1	Probable histone H2A variant 2	Q8S857	Signaling and Cellular Process	−2.16643
2	60S ribosomal protein L30	Q9SDG6	Protein Biosynthesis	−1.79715
3	GTP-binding nuclear protein Ran-2	Q7GD79	Transporter Protein	−1.50517
4	Small ubiquitin-related modifier 1	P55857	Signaling and Cellular Process	−1.22326
5	26S proteasome regulatory subunit 6A homolog	P46465	Translational Modification	−1.16330
6	Mitochondrial outer membrane protein porin 5	Q84P97	Signaling and Cellular Process	−1.03802
7	L-ascorbate peroxidase 1, cytosolic	Q10N21	Amino Acid Biosynthesis	−0.98254
8	Betaine aldehyde dehydrogenase 1	O24174	Amino Acid Biosynthesis	−0.95400
9	Chitinase 2	Q7DNA1	Plant Defense	−0.94951
10	60S ribosomal protein L37a-2	P0DKK2	Protein Biosynthesis	−0.92619
11	Histone H3.3	Q0JCT1	Signaling and Cellular Process	−0.85296
12	Tubulin alpha-1 chain	P28752	Signaling and Cellular Process	−0.82358
13	Probable aldo-keto reductase 2	Q7XT99	N/A	−0.79303
14	Tripeptidyl-peptidase 2	Q6ESI7	Translational Modification	−0.78030
15	5-methyltetrahydropteroyltriglutamate–homocysteine methyltransferase 1	Q2QLY5	Amino Acid Biosynthesis	−0.75822
16	Expansin-A7	Q852A1	Plant Defense	−0.72968
17	Plasma membrane ATPase	Q7XPY2	Transporter Protein	−0.65824

*The list is sorted in descending order in accordance to decreased and then increased in protein abundance of each protein*.

*N/A, not available*.

Essential nutrients in plant can be classified into macro- or micronutrients. Results obtained in this study revealed that a callus treated with optimum PF-68 concentration had higher amounts of macronutrients (K, Mg, and Ca) and micronutrients (Fe, Zn, Cu, and Mn) compared with the control (Table 1). The increase in nutrient uptake observed may be due to enhanced membrane permeability in response to PF-68. Among these plant nutrients tested, K is an essential nutrient and plays a vital role in plant growth and development, which is supported by the highest amount of K uptake in optimum PF-68 concentration. It is involved in biochemical processes, including protein synthesis and carbohydrate metabolism (Wang et al., 2013). Faust and Schubert (2016) previously reported that protein and sugar contents were significantly reduced in sugar beet due to K deficiency. In addition, accumulation of protein precursors, such as amino acids and amides, was also observed, suggesting that K deficiency can inhibit protein synthesis. Zelelew et al. (2016) also reported that increasing the K level had significant effects on plants, whereby plant height, aerial stem number, and number of leaves per plant were increased in a potato variety they studied. Thus, maintaining a balance of the K level in plants is crucial in plant growth and development. Stunted growth, poor root system, lodging, yield reduction, and yellowing of leaf were also common phenomena that occurred when plants had K deficiency (Wang et al., 2013). Furthermore, K deficiency increased plant susceptibility to various diseases and pest infestation, rendering plants to be more susceptible to damage under various stress conditions (Hasanuzzaman et al., 2018). Based on the results obtained in Table 1, the enhanced nutrients uptake, particularly K, could be one of the effects of PF-68, which contributed to enhanced the callus proliferation rate of MR 219.

Secondary plant metabolites are numerous chemical compounds produced by the plant cells through metabolic pathways derived from the primary metabolic pathways. Phenolic and flavonoid compounds are the largest groups of secondary metabolites in plants, and they play important roles in mediating plant response to biotic and abiotic stresses (Park et al., 2018). In this study, an increase in the abundance of PAL proteins (Table 3 and Supplementary Table 4) and high PAL activity (Figure 3A) were recorded in a callus treated with high PF-68 concentration. PAL catalyzes the first reaction in the biosynthesis of ammonia and trans-cinnamate from phenylalanine. The product is then further transformed into a wide variety of phenylpropanoid natural products, including phenolic, flavonoid, lignin, and phytoalexins (Jun et al., 2018). PAL is crucial in plant growth and development as it is responsible for producing secondary metabolites in response to environmental cues, including UV irradiation, exposure to heavy metals, infection, wounding, low temperatures, and low levels of nitrogen, phosphate, or ions (Zhang and Liu, 2015). In the present study, the increase in PAL activity implied that secondary metabolite biosynthesis was enhanced in a callus treated with a high PF-68 concentration. Notably, the increasing appearance of brown calli observed at a high concentration of PF-68 could be associated with the accumulation of secondary metabolites biosynthesis as well (Figure 1B).

Lignin is a phenolic complex polymer involved in plant growth and development as one of the major components of a secondary wall. It also provides mechanical strength to the cell wall and plays an important role in a defense mechanism against biotic and abiotic stresses (Vanholme et al., 2010). Enhanced cell wall lignification has been widely reported in plants when exposed to environmental stresses. For example, plant adaptation toward salt stress resulted in a significant increase in lignin content in the vascular tissues and thickened cell wall (Chun et al., 2019). In this study, cinnamyl alcohol dehydrogenase (CAD) protein was found to be exclusive in a callus treated with high PF-68 concentration as compared with optimum PF-68 concentration (Supplementary Table 4). This CAD has been widely characterized, and it is known to play a role in lignin biosynthesis *via* the conversion of phenylpropenyl aldehydes to alcohols (Ma et al., 2018). Suppression of CAD was demonstrated to reduce the lignin content and alter the lignin composition in switchgrass (Fu et al., 2011). Based on these results, the presence of CAD protein exclusive to a callus treated with high PF-68 concentration suggested that lignin biosynthesis was enhanced in response to increasing stress induced by high PF-68 concentration.

Reactive oxygen species (ROS) is one of the by-products of aerobic metabolism. Peroxidation of lipid membrane is one of the damaging effects of ROS. During the membrane lipid peroxidation process, ROS removes electrons from the lipids in the cell membrane, disrupting cell membrane system of the plant. Enhancement in membrane lipid peroxidation caused an increase in membrane permeability of the plant, which eventually caused electrolytes leakage in a plant cell (Campo et al., 2014). The results obtained in this study showed that enhanced MDA content was recorded in 0.10% PF-68 (Figure 3B). MDA is a major product of lipid peroxidation, and it reflects the degree of lipid peroxidation in plant cells in response to stress (Song et al., 2016). Similar significant levels of lipid peroxidation were detected in forest trees (Zhou et al., 2014) and rapeseed (Jin et al., 2010) in response to abiotic stresses. A significant increase in MDA levels indicates severe oxidative damage occurring in the plant cell membrane. However, studies have found that lipid peroxidation is a common phenomenon that occurred in plant cells when subjected to stress. MDA was often used as a marker to determine the physiological status of the plant during plant growth (Talbi et al., 2015; Kong et al., 2016). In this study, a high level of MDA was detected at a high concentration of PF-68, which signified oxidative damage in the cell membrane. This observation is able to prove the previous hypothesis made on high PF-68 concentration, whereby high PF-68 concentration induced detrimental and irreversible changes in the plasma membrane in plant cells (Curtis and Mirkov, 2012; Irshad et al., 2018).

In line with the raised MDA content, an increase in protein abundance involved in the antioxidant defense system, such as peroxidase protein and peroxiredoxin proteins, was also detected in a callus treated with high PF-68 concentration (Table 4 and Supplementary Table 4). Consistently, the biochemical assessment revealed similar high peroxidase activity in a callus treated with high PF-68 concentration (Figure 3C). Low levels of ROS detected are common in plants as ROS is a by-product of plant aerobic metabolism. Moreover, ROS serves as an important signaling molecule to regulate physiological processes (Wang et al., 2016). However, the accumulation of ROS may cause excessive damage toward macromolecules and plant cells, which eventually triggers a hypersensitive response and programmed cell death (PCD) in plant cells (Konieczny et al., 2014). The excessive production of ROS is often facilitated by increasing stress response (Konieczny et al., 2014). In order to regulate ROS intracellular levels in plants, ROS scavenging enzymes, such as catalase, peroxiredoxin, and peroxidase are required (Chou et al., 2012). The increased synthesis of peroxidase and peroxiredoxin proteins detected in the callus treated with high PF-68 concentration showed that the regulation of ROS was enhanced in response to increasing oxidative stress, induced by high PF-68 concentration.

**Table 4:** Top 20 proteins showing significant abundance difference (together with their accession numbers) when compared between optimum and high PF-68 concentrations.

**No**	**Protein name**	**Uniprot accession no**.	**General function**	**Difference in protein abundance**
**Up-regulated Proteins found in 0.04% PF-68 when compared to high (0.10% PF-68) concentration**				
1	Alpha-amylase isozyme 3A	P27932	Carbohydrate Metabolism	3.01968
2	Alcohol dehydrogenase class-3	Q0DWH1	Carbohydrate Metabolism	1.65944
3	Elongation factor 1-delta 1	Q40680	Protein Biosynthesis	1.63954
4	Obg-like ATPase 1	Q6Z1J6	Stress Response	1.28262
5	GDP-mannose 3,5-epimerase 2	Q2R1V8	Secondary Metabolites Biosynthesis	1.26883
6	Cysteine proteinase inhibitor 4	Q5N806	Plant Defense	1.24385
7	Small ubiquitin-related modifier 1	P55857	Signaling and Cellular Process	1.20008
8	5-methyltetrahydropteroyltriglutamate–homocysteine methyltransferase 1	Q2QLY5	Amino Acid Biosynthesis	1.19441
9	Thioredoxin-like protein CXXS1	Q0J9V5	Translational Modification	1.11076
10	Spermidine synthase 1	Q9SMB1	Amino Acid Biosynthesis	1.09836
11	60S ribosomal protein L9	P49210	Protein Biosynthesis	1.04088
12	60S ribosomal protein L30	Q9SDG6	Protein Biosynthesis	1.02808
13	Nucleosome assembly protein 1;1	Q5VND6	Translational Modification	0.99218
14	Nucleosome assembly protein 1;2	Q53WK4	Translational Modification	0.95744
15	Elongation factor 1-gamma 2	Q6YW46	Protein Biosynthesis	0.95154
16	Tubulin alpha-1 chain	P28752	Signaling and Cellular Process	0.93020
17	Histone H3.2	Q2RAD9	Signaling and Cellular Process	0.90356
18	Cysteine proteinase inhibitor 10	P0C579	Plant Defense	0.87749
19	Tubulin beta-3 chain	Q40665	Signaling and Cellular Process	0.79064
20	6-phosphogluconate dehydrogenase, decarboxylating 2, chloroplastic	Q2R480	Carbohydrate Metabolism	0.77394
**Down-regulated Proteins found in 0.04% PF-68 when compared to high (0.10% PF-68) concentration**				
1	Protein argonaute 1A	Q6EU14	Signaling and Cellular Process	−1.91006
2	12-oxophytodienoate reductase 1	Q84QK0	Plant Defense	−1.24963
3	Peroxidase 2	Q0D3N0	Stress Response	−1.22906
4	Strigolactone esterase D14	Q10QA5	N/A	−1.21814
5	Non-specific lipid-transfer protein 3	Q2QYL3	Plant Defense	−1.16138
6	Glutamate dehydrogenase 2, mitochondrial	Q33E23	Amino Acid Biosynthesis	−1.15802
7	Proteasome subunit alpha type-2	Q10KF0	Translational Modification	−0.96022
8	60S ribosomal protein L5-2	Q8L4L4	Protein Biosynthesis	−0.91828
9	Putative 12-oxophytodienoate reductase 5	Q69TI0	Secondary Metabolites Biosynthesis	−0.90667
10	Profilin LP04	Q5VMJ3	Signaling and Cellular Process	−0.81335
11	Neutral ceramidase	Q0JL46	Lipid Metabolism	−0.80000
12	Arginase 1, mitochondrial	Q7X7N2	Amino Acid Biosynthesis	−0.75186
13	Peroxiredoxin-2C	Q9FR35	Stress Response	−0.74656
14	Ubiquitin-fold modifier 1	Q94DM8	Translational Modification	−0.72888
15	Protein SPIRAL1-like 3	Q2QQ99	Signaling and Cellular Process	−0.69104
16	Succinate-semialdehyde dehydrogenase, mitochondrial	B9F3B6	Carbohydrate Metabolism	−0.68819
17	ATP synthase subunit beta, mitochondrial	Q01859	Transporter Protein	−0.68358
18	Elongation factor Ts, mitochondrial	Q6ZJS7	Protein Biosynthesis	−0.67611
19	Cupincin	Q852L2	N/A	−0.66449
20	Betaine aldehyde dehydrogenase 2	Q84LK3	Stress Response	−0.66356

*The list is sorted in descending order in accordance to decreased and then increased in protein abundance of each protein*.

*N/A, not available*.

Aside from protein abundance in plants, esterase could be used to determine cell viability. Several studies have utilized the changes in esterase activity as a marker to study the growth characteristics and cell viability in plants (Amano et al., 2003; Tamás et al., 2005; Víteček et al., 2007). In a way, a decrease in esterase activity in plants corresponds with the decrease in cell viability in plants. Besides, esterase activity can also act as an excellent bioindicator toward environmental stresses (Radić and Pevalek-Kozlina, 2010). In the present study, the decrease in esterase activity in a high concentration of PF-68 could be associated with the decrease in the calli cells viability. This association was supported by the increasing number of black calli recorded at a high concentration of PF-68 (Figure 1B). The decrease in cell viability was most probably due to the accumulation of ROS (Figure 2C) and the incapability of the ROS scavenging system to cope with the increasing stress induced by high concentration of PF-68, which eventually triggered PCD in the callus cells (Figure 3D).

## Conclusions

**Figure 5:**
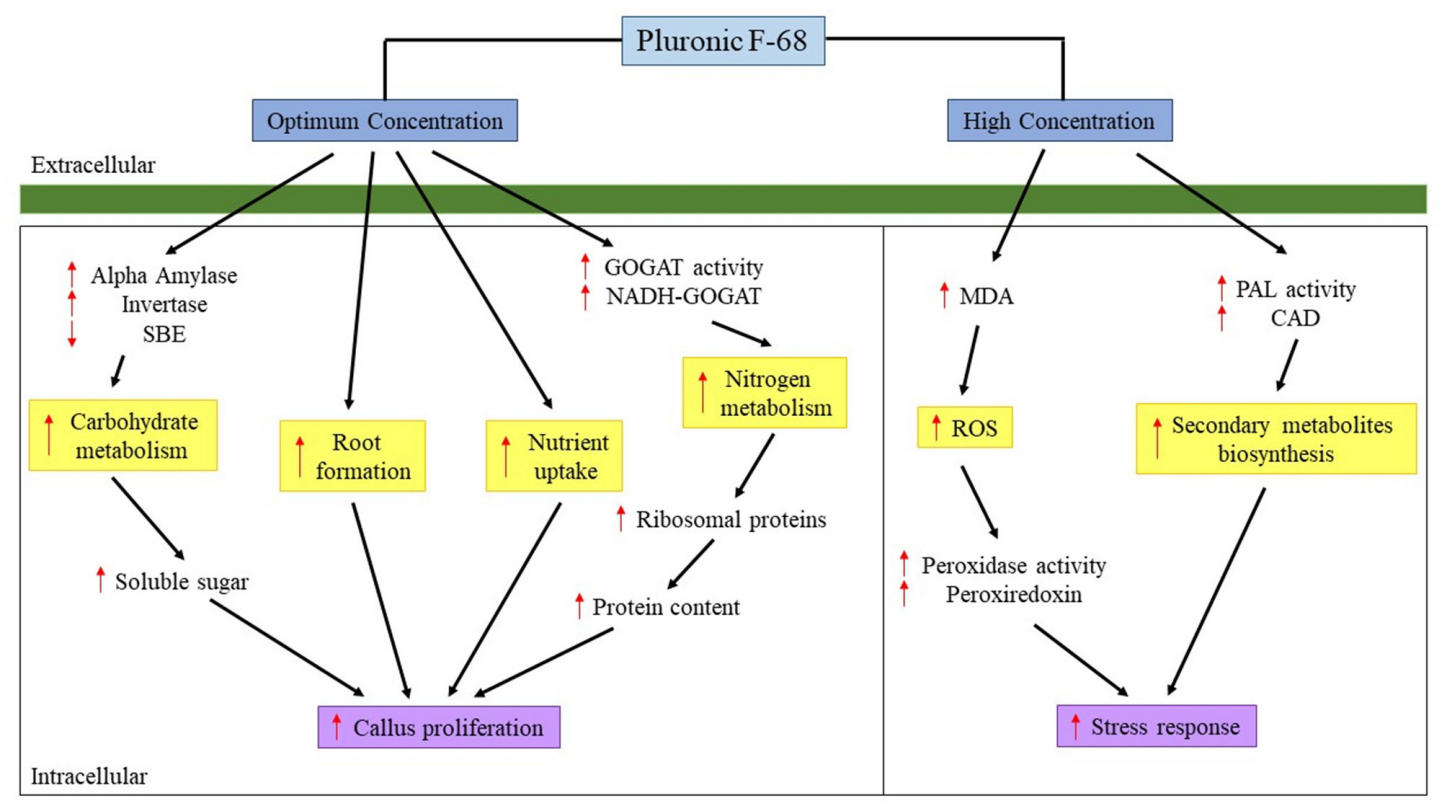
Mechanism proposed on the roles of PF-68 in callus proliferation. CAD, cinnamyl alcohol dehydrogenase; GOGAT, glutamate synthase; MDA, malondialdehyde; NADH-GOGAT, NADH-dependent glutamate synthase; PAL, phenylalanine ammonia lyase; ROS, reactive oxygen species; SBE, starch branching enzyme; ↑, increased; ↓, decreased.

Despite the growth-promoting effects of PF-68 were known, the mechanism governing the growth-promoting effects remains largely fragmented. The present study demonstrates that the growth-promoting effects of PF-68 are independent of a plant hormone. The comparative proteome analysis revealed that optimum PF-68 concentration enhanced callus proliferation of MR 219 cultivar *via* enhanced sugar accumulation and protein biosynthesis. This was evidenced by the increase in the abundance of carbohydrate metabolism-related proteins, ribosomal proteins, and nitrogen metabolism-related proteins (Figure 5). Moreover, optimum PF-68 concentration enhanced nutrients uptake in the callus, particularly K, suggesting a vital role of K in plant growth and development. However, a callus treated with high PF-68 concentration revealed increasing levels of stress response, such that high levels of PAL activity, MDA content, and peroxidase activity were detected (Figure 5). Consistently, an increased abundance of PAL protein and proteins involved in the antioxidant defense system was detected in a callus treated with high PF-68 concentration as well. In addition, the reduced level of esterase activity detected at a callus treated with high PF-68 concentration suggests that increasing stress response eventually triggered PCD. Taken together, the growth-promoting mechanism of PF-68 is concentration-dependent, and incorporation of PF-68 at different concentrations may either promote plant growth or induce stress.

## Data Availability Statement

The original contributions presented in the study are included in the article/Supplementary Materials, further inquiries can be directed to the corresponding author/s. Processed proteomic data can be found in the Supplementary Materials.

## Author Contributions

K-SL and JO-A conceived and designed the experiments. AK performed research, analyzed data, and wrote the manuscript. NM, RS, C-YW, and DL contributed to analytical tools/reagents/funding acquisition. All the authors have read, contributed, and approved the manuscript.

## Conflict of Interest

The authors declare that the research was conducted in the absence of any commercial or financial relationships that could be construed as a potential conflict of interest.

## Supplementary Material

Data Sheet 1 0.04% PF-68.

Data Sheet 2 0.10% PF-68.

Data Sheet 3 Control.

Data Sheet 4 Perseus Proteomic Analysis on Pluronic F-68.

Data Sheet 5 Supplementary Tables and Figures.

Data Sheet 6 Proteomic Data Analysis.

## Abbreviations

CAD: Cinnamyl alcohol dehydrogenase
DW: Dry weight
FW: Fresh weight
GOGAT: Glutamate synthase
MDA: Malondialdehyde
MS: Murashige and Skoog
MSO: Murashige and Skoog without plant growth regulators
PAL: Phenylalanine ammonia lyase
PF-68: Pluronic F-68
ROS: Reactive oxygen species
SBE: Starch branching enzyme.
