# Supplementary material for: Pluronic F-68 Improves Callus Proliferation of Recalcitrant Rice Cultivar via Enhanced Carbon and Nitrogen Metabolism and Nutrients Uptake
Source: Front Plant Sci. 2021 Jun 2;12:667434. doi: 10.3389/fpls.2021.667434 (PMC8207202; doi:10.3389/fpls.2021.667434)
Supplement: Data Sheet 5 — Supplementary Tables and Figures. [file Data_Sheet_5.docx]

**Supplementary Figures**

**Supplementary Figure 1.** Exploratory analysis output of callus exposed to different concentrations of PF-68 via Perseus v1.6.0.7. Comparison between treatment groups, 0% PF-68 and 0.04% PF-68 in terms of **(A)** principle component analysis (PCA), **(B)** multi-scatter plot together with Pearson correlation value and **(C)** volcano plot. Comparison between treatment groups, 0% PF-68 and 0.10% PF-68 in terms of **(D)** PCA, **(E)** multi-scatter plot together with Pearson correlation value and **(F)** volcano plot. Comparison between treatment groups, 0.04% PF-68 and 0.10% PF-68 in terms of **(G)** PCA, **(H)** multi-scatter plot together with Pearson correlation value and **(I)** volcano plot. PCA is attributed to drastic variation of the callus incubated in 0% (□), 0.04% (□) and 0.10% (□) PF-68. Volcano plot indicates up- (□) and down-regulated (□) proteins detected in callus of each treatment groups. Up-regulated and down-regulated proteins indicate proteins with statistical significance of expression at *p*<0.05.

**Supplementary Figure 2.** Normalized relative gene expression of *OsVLN5, OsPYK, OsFD-GOGAT, OsTRX, OsAMY*, and *OsMMT* in calli grown on Murashige and Skoog (MS) medium supplemented with different concentrations (0%, 0.04% and 0.10%) of PF-68 for proteomic confirmation. Data shows the mean of three biological replicates. Asterisks indicate statistically significant at *p*<0.05 compared to control (0% PF-68). Error bars represent standard error mean.

**Supplementary Tables**

**Supplementary Table 1.** Primers used for respective genes together with its function.

**Supplementary Table 2**. Top 100 proteins showing significant abundance difference (together with their accession numbers) in calli subjected to 0.04% PF-68 treatment compared to control (0% PF-68). The list is sorted in descending order in accordance to decreased and then increased in protein abundance of each protein.

**Supplementary Table 3**. Top 98 proteins showing significant abundance difference (together with their accession numbers) in comparison between control calli (0% PF-68) and calli subjected to 0.10% PF-68 treatment. The list is sorted in descending order in accordance to decreased and then increased in protein abundanceof each protein.

**Supplementary Table 4**. Top 100 proteins showing significant abundance difference (together with their accession numbers) in comparison between calli grown on Murashige and Skoog (MS) medium supplemented with either 0.04% or 0.10% PF-68. The list is sorted in descending order in accordance to decreased and then increased in protein abundanceof each protein.

| (A) | (B) | (C) |
| --- | --- | --- |
| 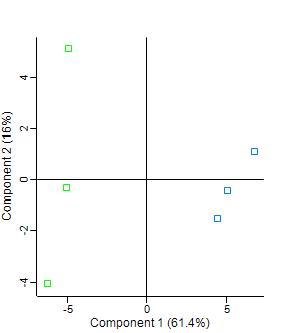 | 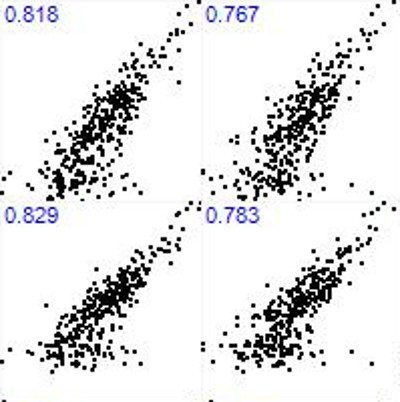 | 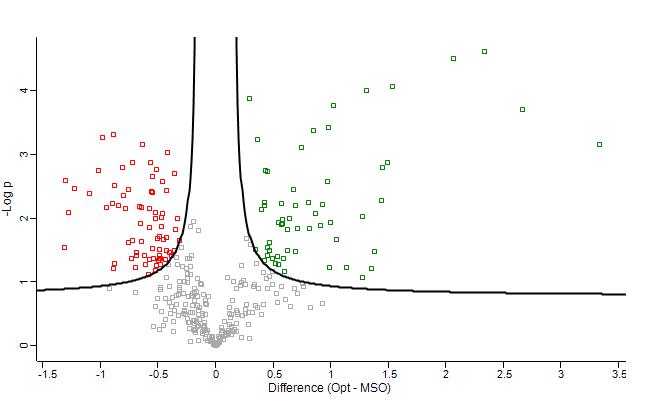 |
| (D) | (E) | (F) |
| 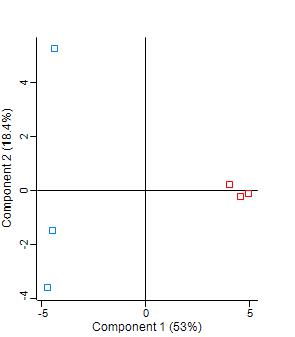 | 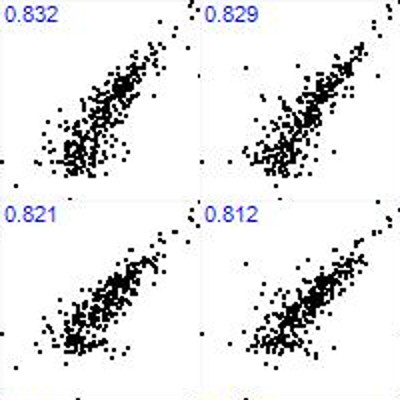 | 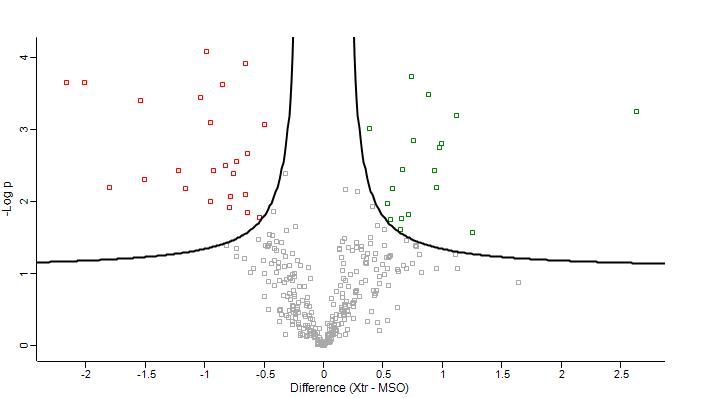 |
| (G) | (H) | (I) |
| 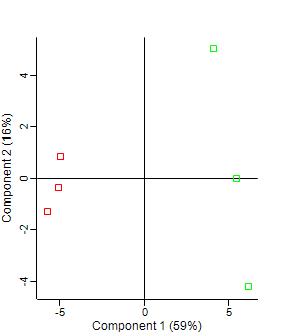 | 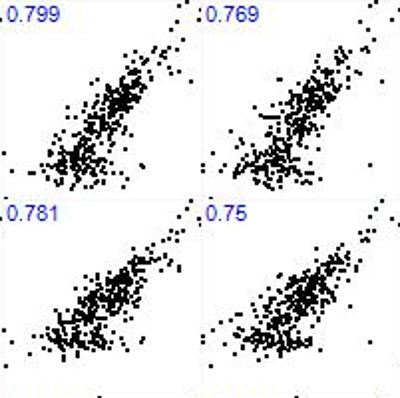 | 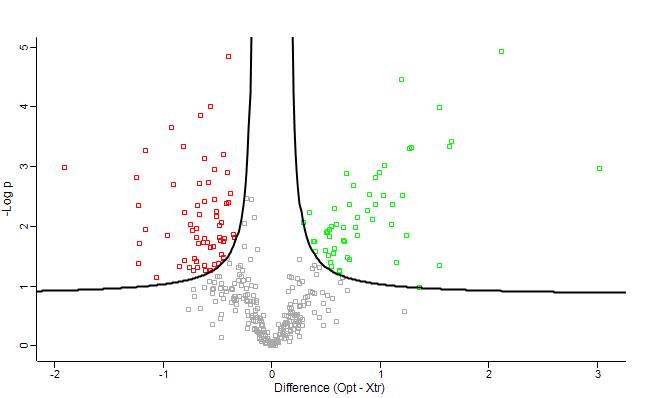 |

**Supplementary Figure 1.** Exploratory analysis output of callus exposed to different concentrations of PF-68 via Perseus v1.6.0.7. Comparison between treatment groups, 0% PF-68 and 0.04% PF-68 in terms of **(A)** principle component analysis (PCA), **(B)** multi-scatter plot together with Pearson correlation value and **(C)** volcano plot. Comparison between treatment groups, 0% PF-68 and 0.10% PF-68 in terms of **(D)** PCA, **(E)** multi-scatter plot together with Pearson correlation value and **(F)** volcano plot. Comparison between treatment groups, 0.04% PF-68 and 0.10% PF-68 in terms of **(G)** PCA, **(H)** multi-scatter plot together with Pearson correlation value and **(I)** volcano plot. PCA is attributed to drastic variation of the callus incubated in 0% (□), 0.04% (□) and 0.10% (□) PF-68. Volcano plot indicates up- (□) and down-regulated (□) proteins detected in callus of each treatment groups. Up-regulated and down-regulated proteins indicate proteins with statistical significance of expression at *p*<0.05.

**Supplementary Figure 2.** Normalized relative gene expression of *OsVLN5, OsPYK, OsFD-GOGAT, OsTRX, OsAMY*, and *OsMMT* in calli grown on Murashige and Skoog (MS) medium supplemented with different concentrations (0%, 0.04% and 0.10%) of PF-68 for proteomic confirmation. Data shows the mean of three biological replicates. Asterisks indicate statistically significant at *p*<0.05 compared to control (0% PF-68). Error bars represent standard error mean.

**Supplementary Table 1.** Primers used for respective genes together with its function.

| Target genes | Primer Sequence 5`–3` | | Gene Function |
| --- | --- | --- | --- |
| *OsVLN5* | Forward | GGAAATATGGCGCATCGAGAAC | Major actin filament stabilizing factor and regulator of actin dynamics (Zhang et al., 2010) |
|  | Reverse | AACCCAGATGCCACTCCTCC |  |
| *OsPYK* | Forward | TCAACCAACGCTACAAATGAATCC | Involved in synthesis of pyruvate and ATP in glycolysis pathway (Valentini et al., 2000) |
|  | Reverse | CTTCACAACCGAGGAATCTCCCAC |  |
| *OsFD-GOGAT* | Forward | CTCGCCTTCTGTGGAAGTGT | Plays central role in the regulation of nitrogen assimilation along with glutamine synthetase in GS/GOGAT cycle (Konishi et al., 2014) |
|  | Reverse | GAGAACGCCTTCACCCAGAA |  |
| *OsTRX* | Forward | TAGTGCTGAGCGATTGTGGA | Involved in redox signaling and able to function as antioxidants by facilitating the reduction of other proteins (Mata-Pérez and Spoel, 2014) |
|  | Reverse | GGCACAGTAGCAGACGATGT |  |
| *OsAMY* | Forward | CCCTCTGGAAAGTACCTCTTTG | Catalyzes the hydrolysis of starch into sugar (Zeeman et al., 2010) |
|  | Reverse | AGAGACTCAAGGGTAGTGAGAG |  |
| *OsMMT* | Forward | CCCTCTGGAAAGTACCTCTTTG | Catalyzes the transfer of a methyl group from 5-methyltetrahydrofolate to homocysteine resulting in methionine formation (Eckermann et al., 2000) |
|  | Reverse | AGAGACTCAAGGGTAGTGAGAG |  |
| Housekeeping genes | Primer Sequence 5`–3` | |  |
| *OsCYC* | Forward | GGTGTCACTCATGACTTCTG | Involved in a wide variety of processes including protein trafficking and maturation, receptor complex stabilization, receptor signaling, RNA processing, and spliceosome assembly (Kumari et al., 2013) |
|  | Reverse | GCCCATCCGAAACGATAC |  |
| *OsUBQ5* | Forward | TAGGCGTAGGCTCCTGTTCT | Involved in protein degradation via the proteasome (Sharma et al., 2016) |
|  | Reverse | ACAGAGGTGATGCTAAGGTGT |  |

**Supplementary Table 2**. Top 100 proteins showing significant abundance difference (together with their accession numbers) in calli subjected to 0.04% PF-68 treatment compared to control (0% PF-68). The list is sorted in descending order in accordance to decreased and then increased in protein abundance of each protein.

| No | Protein Name | | Uniprot Accession No. | General Function | Difference in Protein Abundance |
| --- | --- | --- | --- | --- | --- |
| Up-regulated proteins found in 0.04% PF-68 when compared to control (0% PF-68) | | | | | |
| 1 | Alpha-amylase isozyme 3A |  | P27932 | Carbohydrate Metabolism | 3.33631 |
| 2 | Heat shock 70 kDa protein BIP4 |  | Q75HQ0 | Stress Response | 2.66305 |
| 3 | 60S ribosomal protein L10a |  | B7F845 | Protein Biosynthesis | 2.33374 |
| 4 | Non-specific lipid-transfer protein 1 |  | Q0IQK9 | Transporter Protein | 2.07060 |
| 5 | Obg-like ATPase 1 |  | Q6Z1J6 | Stress Response | 1.53549 |
| 6 | GDP-mannose 3,5-epimerase 2 |  | Q2R1V8 | Secondary Metabolites Biosynthesis | 1.49322 |
| 7 | Cysteine proteinase inhibitor 4 |  | Q5N806 | Plant Defense | 1.44843 |
| 8 | Actin-2 |  | A3C6D7 | Signaling and Cellular Process | 1.43792 |
| 9 | Germin-like protein 3-6 |  | Q851K1 | Plant Defense | 1.38362 |
| 10 | Nucleosome assembly protein 1;1 |  | Q5VND6 | Translational Modification | 1.31477 |
| 11 | Cyanate hydratase |  | Q9FWK4 | Nitrogen Metabolism | 1.28015 |
| 12 | 40S ribosomal protein S21 |  | P35687 | Protein Biosynthesis | 1.05259 |
| 13 | Proteasome subunit alpha type-7-B |  | Q0J006 | Protein Biosynthesis | 1.02611 |
| 14 | Elongation factor 1-delta 1 |  | Q40680 | Protein Biosynthesis | 0.99770 |
| 15 | Ferredoxin--NADP reductase, root isozyme, chloroplastic |  | P41345 | Transporter Protein | 0.98280 |
| 16 | Histone H3.2 |  | Q2RAD9 | Signaling and Cellular Process | 0.96914 |
| 17 | Cinnamyl alcohol dehydrogenase 7 |  | Q0JA75 | Phenylpropanoid Biosynthesis | 0.92934 |
| 18 | Actin-7 |  | P0C540 | Signaling and Cellular Process | 0.91432 |
| 19 | Thioredoxin-like protein CXXS1 |  | Q0J9V5 | Translational Modification | 0.86712 |
| 20 | Delta-aminolevulinic acid dehydratase, chloroplastic |  | Q5Z8V9 | Secondary Metabolites Biosynthesis | 0.84856 |
| 21 | 60S ribosomal protein L9 |  | P49210 | Protein Biosynthesis | 0.81589 |
| 22 | Glutaredoxin-C8 |  | Q0DAE4 | Transporter Protein | 0.80580 |
| 23 | Eukaryotic initiation factor 4A-1 |  | P35683 | Protein biosynthesis | 0.74344 |
| 24 | Ras-related protein RIC2 |  | P40393 | Signaling and Cellular Process | 0.71503 |
| 25 | Alcohol dehydrogenase class-3 |  | Q0DWH1 | Carbohydrate Metabolism | 0.69356 |
| 26 | Phospho-2-dehydro-3-deoxyheptonate aldolase 2, chloroplastic |  | Q75W16 | Amino Acid Biosynthesis | 0.69057 |
| 27 | Non-specific lipid transfer protein-like 1 |  | Q6ASY2 | Transporter Protein | 0.67812 |
| 28 | Nucleosome assembly protein 1;2 |  | Q53WK4 | Translational Modification | 0.64473 |
| 29 | Glutamate synthase 1 [NADH], chloroplastic |  | Q0JKD0 | Amino Acid Biosynthesis | 0.62797 |
| 30 | Probable calcium-binding protein CML7 |  | Q84VG0 | Plant Pathogen Interaction | 0.62097 |
| Exclusive proteins found in 0.04% PF-68 when compared to control (0% PF-68) | | | | | |
| 31 | 1,2-dihydroxy-3-keto-5-methylthiopentene dioxygenase 2 |  | Q10RE5 | Amino Acid Biosynthesis | N/A |
| 32 | 17.9 kDa class I heat shock protein |  | Q84Q77 | Stress Response | N/A |
| 33 | 26S proteasome regulatory subunit 7B |  | P0DKK0 | Protein Biosynthesis | N/A |
| 34 | 2-C-methyl-D-erythritol 2,4-cyclodiphosphate synthase, chloroplastic | | Q6EPN6 | Secondary Metabolites Biosynthesis | N/A |
| 35 | 40S ribosomal protein S13-1 |  | Q69UI2 | Protein Biosynthesis | N/A |
| 36 | Beta-glucosidase 31 |  | B7F7K7 | Phenylpropanoid Biosynthesis | N/A |
| 37 | Beta-glucosidase 7 |  | Q75I93 | Phenylpropanoid Biosynthesis | N/A |
| 38 | Calcium-dependent protein kinase 28 |  | Q2QX45 | Plant Pathogen Interaction | N/A |
| 39 | Carbamoyl-phosphate synthase small chain, chloroplastic |  | Q6YV23 | Amino Acid Biosynthesis | N/A |
| 40 | Cinnamoyl-CoA reductase 1 |  | Q6K9A2 | Phenylpropanoid Biosynthesis | N/A |
| 41 | Cytosolic invertase 1 |  | Q69T31 | Carbohydrate Metabolism | N/A |
| 42 | Elongation factor 1-delta 2 |  | Q40682 | Protein Biosynthesis | N/A |
| 43 | Enoyl-[acyl-carrier-protein] reductase [NADH] 2, chloroplastic | | Q6H5J0 | Lipid Metabolism | N/A |
| 44 | Eukaryotic translation initiation factor isoform 4G-1 |  | Q84PB3 | Protein Biosynthesis | N/A |
| 45 | Glucosidase 2 subunit beta |  | Q5NBP9 | Plant Defense | N/A |
| 46 | Glutamine synthetase cytosolic isozyme 1-3 |  | Q4W8D0 | Amino Acid Biosynthesis | N/A |
| 47 | Homogentisate 1,2-dioxygenase |  | Q5VRH4 | Amino Acid Biosynthesis | N/A |
| 48 | Inositol-3-phosphate synthase 1 |  | O64437 | Carbohydrate Metabolism | N/A |
| 49 | Momilactone A synthase |  | Q7FAE1 | Secondary Metabolites Biosynthesis | N/A |
| 50 | Phosphomannomutase |  | Q7XPW5 | Carbohydrate Metabolism | N/A |
| Down-regulated proteins found in 0.04% PF-68 when compared to control (0% PF-68) | | | | | |
| 51 | Villin-5 |  | Q0J716 | Signaling and Cellular Process | -1.31358 |
| 52 | Elongation factor Ts, mitochondrial |  | Q6ZJS7 | Protein Biosynthesis | -1.30464 |
| 53 | Peroxidase 2 |  | Q0D3N0 | Phenylpropanoid Biosynthesis | -1.27717 |
| 54 | Protein argonaute 1A |  | Q6EU14 | N/A | -1.22130 |
| 55 | Neutral ceramidase |  | Q0JL46 | Lipid Metabolism | -1.09147 |
| 56 | Peroxiredoxin-2C |  | Q9FR35 | N/A | -1.01497 |
| 57 | Glutamate dehydrogenase 2, mitochondrial |  | Q33E23 | Amino Acid Biosynthesis | -0.97888 |
| 58 | 12-oxophytodienoate reductase 1 |  | Q84QK0 | Lipid Metabolism | -0.94814 |
| 59 | NADP-dependent malic enzyme, chloroplastic |  | P43279 | Carbohydrate Metabolism | -0.89326 |
| 60 | ATP synthase subunit alpha, mitochondrial |  | P0C522 | N/A | -0.88513 |
| 61 | Putative 12-oxophytodienoate reductase 5 |  | Q69TI0 | Lipid Metabolism | -0.87959 |
| 62 | Profilin LP04 |  | Q5VMJ3 | Signaling and Cellular Process | -0.84333 |
| 63 | 60S acidic ribosomal protein P0 |  | P41095 | Protein Biosynthesis | -0.80776 |
| 64 | ATP synthase subunit beta, mitochondrial |  | Q01859 | N/A | -0.79720 |
| 65 | Actin-depolymerizing factor 2 |  | Q9AY76 | Signaling and Cellular Process | -0.78577 |
| 66 | 2-Cys peroxiredoxin BAS1, chloroplastic |  | Q6ER94 | Plant Defense | -0.75732 |
| 67 | Oryzain gamma chain |  | P25778 | Protein Biosynthesis | -0.75668 |
| 68 | Clathrin heavy chain 2 |  | Q2QYW2 | N/A | -0.73074 |
| 69 | Ubiquitin-fold modifier 1 |  | Q94DM8 | Translational Modification | -0.72169 |
| 70 | L-ascorbate peroxidase 1, cytosolic |  | Q10N21 | Amino Acid Biosynthesis | -0.72137 |
| 71 | 60S ribosomal protein L30 |  | Q9SDG6 | Protein Biosynthesis | -0.69000 |
| 72 | Probable 6-phosphogluconolactonase 4, chloroplastic |  | Q69NG5 | Carbohydrate Metabolism | -0.68206 |
| 73 | Alpha-amylase isozyme 3D |  | P27933 | Carbohydrate Metabolism | -0.66052 |
| 74 | 1,4-alpha-glucan-branching enzyme, chloroplastic/amyloplastic | | Q01401 | N/A | -0.65548 |
| 75 | Aminopeptidase M1-B |  | Q0J5V5 | Protein Biosynthesis | -0.64088 |
| Exclusive proteins found in 0% PF-68 when compared to optimum (0.04% PF-68) concentration | | | | | |
| 76 | 60S ribosomal protein L37a-2 |  | P0DKK2 | Protein Biosynthesis | N/A |
| 77 | Actin-related protein 4 |  | Q6ZJW9 | Signaling and Cellular Process | N/A |
| 78 | Asparagine synthetase [glutamine-hydrolyzing] 1 |  | Q10MX3 | Amino Acid Biosynthesis | N/A |
| 79 | ATP phosphoribosyltransferase, chloroplastic |  | Q10S55 | Amino Acid Biosynthesis | N/A |
| 80 | Beta-fructofuranosidase, insoluble isoenzyme 5 |  | Q56UD1 | Carbohydrate Metabolism | N/A |
| 81 | Chitinase 2 |  | Q7DNA1 | Plant Defense | N/A |
| 82 | Cinnamyl alcohol dehydrogenase 2 |  | Q6ZHS4 | Phenylpropanoid Biosynthesis | N/A |
| 83 | Deoxyuridine 5'-triphosphate nucleotidohydrolase |  | Q10FF9 | Nucleotide Metabolism | N/A |
| 84 | Dihydroorotase, mitochondrial |  | Q0JJD1 | Nucleotide Metabolism | N/A |
| 85 | Glutaredoxin-C6 |  | P55142 | Transporter Protein | N/A |
| 86 | Histidinol dehydrogenase, chloroplastic |  | Q5NAY4 | Amino Acid Biosynthesis | N/A |
| 87 | Histone H2B.5 |  | Q94JE1 | Signaling and Cellular Process | N/A |
| 88 | IAA-amino acid hydrolase ILR1-like 7 |  | Q8H3C9 | Protein Biosynthesis | N/A |
| 89 | Mitochondrial outer membrane protein porin 3 |  | Q7F4F8 | Transporter Protein | N/A |
| 90 | Mitochondrial outer membrane protein porin 5 |  | Q84P97 | Transporter Protein | N/A |
| 91 | NPL4-like protein |  | Q9AS33 | Translational Modification | N/A |
| 92 | Phytanoyl-CoA dioxygenase 1 |  | Q10E49 | Lipid Metabolism | N/A |
| 93 | Plasma membrane ATPase |  | Q7XPY2 | Transporter Protein | N/A |
| 94 | Porphobilinogen deaminase, chloroplastic |  | Q6H6D2 | Secondary Metabolites Biosynthesis | N/A |
| 95 | Probable (S)-ureidoglycine aminohydrolase |  | Q7F1K9 | Nucleotide Metabolism | N/A |
| 96 | Probable 5'-adenylylsulfate reductase 1, chloroplastic |  | Q6Z4A7 | Stress Response | N/A |
| 97 | Probable glucan 1,3-alpha-glucosidase |  | B9F676 | Plant Defense | N/A |
| 98 | Probable histone H2A variant 2 |  | Q8S857 | Signaling and Cellular Process | N/A |
| 99 | Probable histone H2A.1 |  | Q6ZL43 | Signaling and Cellular Process | N/A |
| 100 | Protein IN2-1 homolog B |  | Q8H8U5 | Amino Acid Biosynthesis | N/A |

N/A indicates not available

**Supplementary Table 3**. Top 98 proteins showing significant abundance difference (together with their accession numbers) in comparison between control calli (0% PF-68) and calli subjected to 0.10% PF-68 treatment. The list is sorted in descending order in accordance to decreased and then increased in protein abundance of each protein.

| No | Protein Name |  | Uniprot Accession No. | General Function | Difference in Protein Abundance |
| --- | --- | --- | --- | --- | --- |
| Up-regulated proteins found in 0.10% PF-68 when compared to control (0% PF-68) | | | | | |
| 1 | Probable histone H2A.1 |  | Q6ZL43 | Signaling and Cellular Process | 2.63844 |
| 2 | Thioredoxin Y, chloroplastic |  | Q5JMR9 | Translational Modification | 1.12346 |
| 3 | Ferredoxin-dependent glutamate synthase, chloroplastic |  | Q69RJ0 | Amino Acid Biosynthesis | 0.99525 |
| 4 | Ferredoxin--NADP reductase, root isozyme, chloroplastic |  | P41345 | N/A | 0.97960 |
| 5 | Probable L-ascorbate peroxidase 6, chloroplastic/mitochondrial | | P0C0L1 | Amino Acid Biosynthesis | 0.95538 |
| 6 | Glutamate synthase 1 [NADH], chloroplastic |  | Q0JKD0 | Amino Acid Biosynthesis | 0.93512 |
| 7 | Chaperone protein ClpC1, chloroplastic |  | Q7F9I1 | Translational Modification | 0.88085 |
| 8 | Cupincin |  | Q852L2 | N/A | 0.75954 |
| 9 | Phenylalanine ammonia-lyase |  | P14717 | Secondary Metabolites Biosynthesis | 0.74090 |
| 10 | Non-specific lipid-transfer protein 3 |  | Q2QYL3 | Plant Defense | 0.66712 |
| 11 | Putative 12-oxophytodienoate reductase 4 |  | Q69TH8 | Secondary Metabolites Biosynthesis | 0.58307 |
| 12 | Proteasome subunit alpha type-3 |  | Q9LSU0 | Protein Biosynthesis | 0.54185 |
| 13 | 1-Cys peroxiredoxin B |  | P0C5D1 | Stress Response | 0.38496 |
| Exclusive proteins found in 0.10% PF-68 when compared to control (0% PF-68) | | | | | |
| 14 | 60S ribosomal protein L10a |  | B7F845 | Translational Modification | N/A |
| 15 | Actin-2 |  | A3C6D7 | Signaling and Cellular Process | N/A |
| 16 | Actin-7 |  | P0C540 | Signaling and Cellular Process | N/A |
| 17 | Chalcone--flavonone isomerase |  | Q84T92 | Secondary Metabolites Biosynthesis | N/A |
| 18 | Delta-aminolevulinic acid dehydratase, chloroplastic |  | Q5Z8V9 | Secondary Metabolites Biosynthesis | N/A |
| 19 | Enoyl-[acyl-carrier-protein] reductase [NADH] 2, chloroplastic | | Q6H5J0 | Lipid Metabolism | N/A |
| 20 | Germin-like protein 3-6 |  | Q851K1 | N/A | N/A |
| 21 | GTP-binding nuclear protein Ran-1 |  | Q7F7I7 | Transporter Protein | N/A |
| 22 | Heat shock 70 kDa protein BIP4 |  | Q75HQ0 | Stress Response | N/A |
| 23 | Inositol-3-phosphate synthase 1 |  | O64437 | Carbohydrate Metabolism | N/A |
| 24 | Late embryogenesis abundant protein 1 |  | A3AHG5 | Stress Response | N/A |
| 25 | Non-specific lipid-transfer protein 1 |  | Q0IQK9 | Transporter Protein | N/A |
| 26 | Probable 4-coumarate--CoA ligase 3 |  | Q6ETN3 | Secondary Metabolites Biosynthesis | N/A |
| 27 | Probable ureidoglycolate hydrolase |  | Q2QMN7 | Nucleotide Metabolism | N/A |
| 28 | S-adenosylmethionine synthase 1 |  | Q0DKY4 | Secondary Metabolites Biosynthesis | N/A |
| 29 | Tubulin beta-5 chain |  | P46265 | Signaling and Cellular Process | N/A |
| 30 | Ubiquitin-60S ribosomal protein L40-2 |  | P0CH35 | Translational Modification | N/A |
| 31 | UDP-arabinopyranose mutase 3 |  | Q6Z4G3 | Nucleotide Metabolism | N/A |
| Down-regulated proteins found in 0.10% PF-68 when compared to control (0% PF-68) | | | | | |
| 32 | Probable histone H2A variant 2 |  | Q8S857 | Signaling and Cellular Process | -2.16643 |
| 33 | 60S ribosomal protein L30 |  | Q9SDG6 | Protein Biosynthesis | -1.79715 |
| 34 | GTP-binding nuclear protein Ran-2 |  | Q7GD79 | Transporter Protein | -1.50517 |
| 35 | Small ubiquitin-related modifier 1 |  | P55857 | Signaling and Cellular Process | -1.22326 |
| 36 | 26S proteasome regulatory subunit 6A homolog |  | P46465 | Translational Modification | -1.16330 |
| 37 | Mitochondrial outer membrane protein porin 5 |  | Q84P97 | Signaling and Cellular Process | -1.03802 |
| 38 | L-ascorbate peroxidase 1, cytosolic |  | Q10N21 | Amino Acid Biosynthesis | -0.98254 |
| 39 | Betaine aldehyde dehydrogenase 1 |  | O24174 | Amino Acid Biosynthesis | -0.95400 |
| 40 | Chitinase 2 |  | Q7DNA1 | Plant Defense | -0.94951 |
| 41 | 60S ribosomal protein L37a-2 |  | P0DKK2 | Protein Biosynthesis | -0.92619 |
| 42 | Histone H3.3 |  | Q0JCT1 | Signaling and Cellular Process | -0.85296 |
| 43 | Tubulin alpha-1 chain |  | P28752 | Signaling and Cellular Process | -0.82358 |
| 44 | Probable aldo-keto reductase 2 |  | Q7XT99 | N/A | -0.79303 |
| 45 | Tripeptidyl-peptidase 2 |  | Q6ESI7 | Translational Modification | -0.78030 |
| 46 | 5-methyltetrahydropteroyltriglutamate--homocysteine methyltransferase 1 | | Q2QLY5 | Amino Acid Biosynthesis | -0.75822 |
| 47 | Expansin-A7 |  | Q852A1 | Plant Defense | -0.72968 |
| 48 | Plasma membrane ATPase |  | Q7XPY2 | Transporter Protein | -0.65824 |
| 49 | Guanine nucleotide-binding protein subunit beta-like protein A | | P49027 | Signaling and Cellular Process | -0.65239 |
| 50 | Probable nucleoredoxin 1-1 |  | Q7Y0E8 | N/A | -0.49847 |
| Exclusive proteins found in 0% PF-68 when compared to high (0.10% PF-68) concentration | | | | | |
| 51 | 26S proteasome non-ATPase regulatory subunit 6 |  | Q8W425 | Translational Modification | N/A |
| 52 | Acetyl-CoA carboxylase 2 |  | B9FK36 | Carbohydrate Metabolism | N/A |
| 53 | Actin-3 |  | Q10AZ4 | Signaling and Cellular Process | N/A |
| 54 | Actin-depolymerizing factor 2 |  | Q9AY76 | Signaling and Cellular Process | N/A |
| 55 | Actin-related protein 4 |  | Q6ZJW9 | Signaling and Cellular Process | N/A |
| 56 | Alcohol dehydrogenase class-3 |  | Q0DWH1 | Secondary Metabolites Biosynthesis | N/A |
| 57 | Anamorsin homolog 1 |  | Q7XQ97 | Signaling and Cellular Process | N/A |
| 58 | ATP phosphoribosyltransferase, chloroplastic |  | Q10S55 | Amino Acid Biosynthesis | N/A |
| 59 | Beta-fructofuranosidase, insoluble isoenzyme 5 |  | Q56UD1 | Carbohydrate Metabolism | N/A |
| 60 | Beta-galactosidase 15 |  | Q0INM3 | Carbohydrate Metabolism | N/A |
| 61 | Carbamoyl-phosphate synthase large chain, chloroplastic |  | B9EXM2 | Amino Acid Biosynthesis | N/A |
| 62 | Clathrin light chain 1 |  | Q7XKE9 | Transporter Protein | N/A |
| 63 | Coatomer subunit delta-1 |  | Q0DJA0 | Transporter Protein | N/A |
| 64 | DEAD-box ATP-dependent RNA helicase 38 |  | Q10RI7 | Transporter Protein | N/A |
| 65 | Deoxyuridine 5'-triphosphate nucleotidohydrolase |  | Q10FF9 | Nucleotide Metabolism | N/A |
| 66 | Dihydroorotase, mitochondrial |  | Q0JJD1 | Nucleotide Metabolism | N/A |
| 67 | Elongation factor 1-delta 1 |  | Q40680 | Protein Biosynthesis | N/A |
| 68 | Elongation factor 1-gamma 2 |  | Q6YW46 | Protein Biosynthesis | N/A |
| 69 | Elongation factor 1-gamma 3 |  | Q5Z627 | Protein Biosynthesis | N/A |
| 70 | Esterase PIR7B |  | Q0JG99 | N/A | N/A |
| 71 | Germin-like protein 1-1 |  | Q7F731 | N/A | N/A |
| 72 | IAA-amino acid hydrolase ILR1-like 1 |  | Q8S9S4 | Protein Biosynthesis | N/A |
| 73 | Importin subunit alpha-1a |  | Q71VM4 | Plant Pathogen Interaction | N/A |
| 74 | Importin subunit alpha-1b |  | Q9SLX0 | Transporter Protein | N/A |
| 75 | Leukotriene A-4 hydrolase homolog |  | Q84TA3 | Lipid Metabolism | N/A |
| 76 | Methylcrotonoyl-CoA carboxylase subunit alpha, mitochondrial | | Q2QMG2 | Amino Acid Biosynthesis | N/A |
| 77 | Mitochondrial outer membrane protein porin 3 |  | Q7F4F8 | Signaling and Cellular Process | N/A |
| 78 | Neutral ceramidase |  | Q0JL46 | Lipid Metabolism | N/A |
| 79 | Nucleosome assembly protein 1;2 |  | Q53WK4 | Translational Modification | N/A |
| 80 | Peroxisomal membrane protein 11-5 |  | Q5VRJ8 | N/A | N/A |
| 81 | Phytanoyl-CoA dioxygenase 1 |  | Q10E49 | Transporter Protein | N/A |
| 82 | Polygalacturonase inhibitor 1 |  | Q8GT95 | N/A | N/A |
| 83 | Porphobilinogen deaminase, chloroplastic |  | Q6H6D2 | Secondary Metabolites Biosynthesis | N/A |
| 84 | Probable (S)-ureidoglycine aminohydrolase |  | Q7F1K9 | Nucleotide Metabolism | N/A |
| 85 | Probable glucan 1,3-alpha-glucosidase |  | B9F676 | Translational Modification | N/A |
| 86 | Probable methionine--tRNA ligase |  | Q9ZTS1 | Amino Acid Biosynthesis | N/A |
| 87 | Probable N-acetyl-gamma-glutamyl-phosphate reductase, chloroplastic | | Q6AV34 | Secondary Metabolites Biosynthesis | N/A |
| 88 | Probable protein phosphatase 2C 45 |  | Q7XR06 | N/A | N/A |
| 89 | Protein SGT1 homolog |  | Q0JL44 | Plant Pathogen Interaction | N/A |
| 90 | Putative D-cysteine desulfhydrase 1, mitochondrial |  | Q6ZHE5 | Amino Acid Biosynthesis | N/A |
| 91 | Pyruvate dehydrogenase E1 component subunit beta-2, mitochondrial | | Q0J0H4 | Carbohydrate Metabolism | N/A |
| 92 | Succinate dehydrogenase subunit 6, mitochondrial |  | Q6ZCC4 | N/A | N/A |
| 93 | Thioredoxin M2, chloroplastic |  | Q7X8R5 | Transporter Protein | N/A |
| 94 | Thioredoxin-like protein Clot |  | Q5Z9Z3 | Transporter Protein | N/A |
| 95 | TPR repeat-containing thioredoxin TDX |  | Q6ES52 | Transporter Protein | N/A |
| 96 | Tubulin beta-3 chain |  | Q40665 | Signaling and Cellular Process | N/A |
| 97 | Ubiquitin-40S ribosomal protein S27a-1 |  | Q9ARZ9 | Translational Modification | N/A |
| 98 | Ubiquitin-fold modifier-conjugating enzyme 1 |  | Q8S625 | Translational Modification | N/A |

N/A indicates not available

**Supplementary Table 4**. Top 100 proteins showing significant abundance difference (together with their accession numbers) in comparison between calli grown on Murashige and Skoog (MS) medium supplemented with either 0.04% or 0.10% PF-68. The list is sorted in descending order in accordance to decreased and then increased in protein abundance of each protein.

| No | Protein Name |  | Uniprot Accession No. | General Function | Difference in Protein Abundance |
| --- | --- | --- | --- | --- | --- |
| Up-regulated proteins found in 0.04% PF-68 when compared to high (0.10% PF-68) concentration | | | | | |
| 1 | Alpha-amylase isozyme 3A |  | P27932 | Carbohydrate Metabolism | 3.01968 |
| 2 | Alcohol dehydrogenase class-3 |  | Q0DWH1 | Carbohydrate Metabolism | 1.65944 |
| 3 | Elongation factor 1-delta 1 |  | Q40680 | Protein Biosynthesis | 1.63954 |
| 4 | Obg-like ATPase 1 |  | Q6Z1J6 | Stress Response | 1.28262 |
| 5 | GDP-mannose 3,5-epimerase 2 |  | Q2R1V8 | Secondary Metabolites Biosynthesis | 1.26883 |
| 6 | Cysteine proteinase inhibitor 4 |  | Q5N806 | Plant Defense | 1.24385 |
| 7 | Small ubiquitin-related modifier 1 |  | P55857 | Signaling and Cellular Process | 1.20008 |
| 8 | 5-methyltetrahydropteroyltriglutamate--homocysteine methyltransferase 1 | | Q2QLY5 | Amino Acid Biosynthesis | 1.19441 |
| 9 | Thioredoxin-like protein CXXS1 |  | Q0J9V5 | Translational Modification | 1.11076 |
| 10 | Spermidine synthase 1 |  | Q9SMB1 | Amino Acid Biosynthesis | 1.09836 |
| 11 | 60S ribosomal protein L9 |  | P49210 | Protein Biosynthesis | 1.04088 |
| 12 | 60S ribosomal protein L30 |  | Q9SDG6 | Protein Biosynthesis | 1.02808 |
| 13 | Nucleosome assembly protein 1;1 |  | Q5VND6 | Translational Modification | 0.99218 |
| 14 | Nucleosome assembly protein 1;2 |  | Q53WK4 | Translational Modification | 0.95744 |
| 15 | Elongation factor 1-gamma 2 |  | Q6YW46 | Protein Biosynthesis | 0.95154 |
| 16 | Tubulin alpha-1 chain |  | P28752 | Signaling and Cellular Process | 0.93020 |
| 17 | Histone H3.2 |  | Q2RAD9 | Signaling and Cellular Process | 0.90356 |
| 18 | Cysteine proteinase inhibitor 10 |  | P0C579 | Plant Defense | 0.87749 |
| 19 | Tubulin beta-3 chain |  | Q40665 | Signaling and Cellular Process | 0.79064 |
| 20 | 6-phosphogluconate dehydrogenase, decarboxylating 2, chloroplastic | | Q2R480 | Carbohydrate Metabolism | 0.77394 |
| Exclusive proteins found in 0.04% PF-68 when compared to high (0.10% PF-68) concentration | | | | | |
| 21 | 26S proteasome non-ATPase regulatory subunit 6 |  | Q8W425 | Translational Modification | N/A |
| 22 | 26S proteasome regulatory subunit 6A homolog |  | P46465 | Translational Modification | N/A |
| 23 | 26S proteasome regulatory subunit 7B |  | P0DKK0 | Protein Biosynthesis | N/A |
| 24 | 2-C-methyl-D-erythritol 2,4-cyclodiphosphate synthase, chloroplastic | | Q6EPN6 | Secondary Metabolites Biosynthesis | N/A |
| 25 | 40S ribosomal protein S13-1 |  | Q69UI2 | Protein Biosynthesis | N/A |
| 26 | 40S ribosomal protein S21 |  | P35687 | Protein Biosynthesis | N/A |
| 27 | Acetyl-CoA carboxylase 2 |  | B9FK36 | Carbohydrate Metabolism | N/A |
| 28 | Actin-3 |  | Q10AZ4 | Signaling and Cellular Process | N/A |
| 29 | Actin-depolymerizing factor 2 |  | Q9AY76 | Signaling and Cellular Process | N/A |
| 30 | Beta-galactosidase 15 | | Q0INM3 | Carbohydrate Metabolism | N/A |
| 31 | Beta-glucosidase 31 |  | B7F7K7 | Phenylpropanoid Biosynthesis | N/A |
| 32 | Beta-glucosidase 7 |  | Q75I93 | Phenylpropanoid Biosynthesis | N/A |
| 33 | Betaine aldehyde dehydrogenase 1 |  | O24174 | Amino Acid Biosynthesis | N/A |
| 34 | Calcium-dependent protein kinase 28 |  | Q2QX45 | Plant Pathogen Interaction | N/A |
| 35 | Carbamoyl-phosphate synthase large chain, chloroplastic |  | B9EXM2 | Amino Acid Biosynthesis | N/A |
| 36 | Carbamoyl-phosphate synthase small chain, chloroplastic |  | Q6YV23 | Amino Acid Biosynthesis | N/A |
| 37 | Cinnamoyl-CoA reductase 1 |  | Q6K9A2 | Phenylpropanoid Biosynthesis | N/A |
| 38 | Clathrin light chain 1 |  | Q7XKE9 | Transporter Protein | N/A |
| 39 | Coatomer subunit delta-1 |  | Q0DJA0 | Transporter Protein | N/A |
| 40 | Cytosolic invertase 1 |  | Q69T31 | Carbohydrate Metabolism | N/A |
| Down-regulated proteins found in 0.04% PF-68 when compared to high (0.10% PF-68) concentration | | | | | |
| 41 | Protein argonaute 1A |  | Q6EU14 | Signaling and Cellular Process | -1.91006 |
| 42 | 12-oxophytodienoate reductase 1 |  | Q84QK0 | Plant Defense | -1.24963 |
| 43 | Peroxidase 2 |  | Q0D3N0 | Stress Response | -1.22906 |
| 44 | Strigolactone esterase D14 |  | Q10QA5 | N/A | -1.21814 |
| 45 | Non-specific lipid-transfer protein 3 |  | Q2QYL3 | Plant Defense | -1.16138 |
| 46 | Glutamate dehydrogenase 2, mitochondrial |  | Q33E23 | Amino Acid Biosynthesis | -1.15802 |
| 47 | Proteasome subunit alpha type-2 |  | Q10KF0 | Translational Modification | -0.96022 |
| 48 | 60S ribosomal protein L5-2 |  | Q8L4L4 | Protein Biosynthesis | -0.91828 |
| 49 | Putative 12-oxophytodienoate reductase 5 |  | Q69TI0 | Secondary Metabolites Biosynthesis | -0.90667 |
| 50 | Profilin LP04 |  | Q5VMJ3 | Signaling and Cellular Process | -0.81335 |
| 51 | Neutral ceramidase |  | Q0JL46 | Lipid Metabolism | -0.80000 |
| 52 | Arginase 1, mitochondrial |  | Q7X7N2 | Amino Acid Biosynthesis | -0.75186 |
| 53 | Peroxiredoxin-2C |  | Q9FR35 | Stress Response | -0.74656 |
| 54 | Ubiquitin-fold modifier 1 |  | Q94DM8 | Translational Modification | -0.72888 |
| 55 | Protein SPIRAL1-like 3 |  | Q2QQ99 | Signaling and Cellular Process | -0.69104 |
| 56 | Succinate-semialdehyde dehydrogenase, mitochondrial |  | B9F3B6 | Carbohydrate Metabolism | -0.68819 |
| 57 | ATP synthase subunit beta, mitochondrial |  | Q01859 | Transporter Protein | -0.68358 |
| 58 | Elongation factor Ts, mitochondrial |  | Q6ZJS7 | Protein Biosynthesis | -0.67611 |
| 59 | Cupincin |  | Q852L2 | N/A | -0.66449 |
| 60 | Betaine aldehyde dehydrogenase 2 |  | Q84LK3 | Stress Response | -0.66356 |
| 61 | Alpha-amylase |  | P17654 | Carbohydrate Metabolism | -0.65633 |
| 62 | 1,4-alpha-glucan-branching enzyme, chloroplastic/amyloplastic |  | Q01401 | N/A | -0.63048 |
| 63 | Sucrose-phosphatase 2 |  | Q6YXW6 | Carbohydrate Metabolism | -0.61563 |
| 64 | Electron transfer flavoprotein subunit alpha, mitochondrial |  | Q75LJ3 | Signaling and Cellular Process | -0.61523 |
| 65 | 60S ribosomal protein L11 |  | Q0DK10 | Protein Biosynthesis | -0.61489 |
| 66 | Proteasome subunit beta type-1 |  | O64464 | Translational Modification | -0.59301 |
| 67 | 60S acidic ribosomal protein P0 |  | Q42971 | Protein Biosynthesis | -0.58574 |
| 68 | Enolase |  | Q0IWL9 | Glycolysis / Gluconeogenesis | -0.56410 |
| 69 | Monothiol glutaredoxin-S11 |  | Q5VRJ8 | Translational Modification | -0.56286 |
| 70 | Peroxisomal membrane protein 11-5 |  | P42862 | N/A | -0.53794 |
| 71 | Glucose-6-phosphate isomerase, cytosolic A |  | Q6K9N6 | Carbohydrate Metabolism | -0.52633 |
| 72 | Succinate--CoA ligase [ADP-forming] subunit beta, mitochondrial |  | Q9LI00 | Carbohydrate Metabolism | -0.52536 |
| 73 | 6-phosphogluconate dehydrogenase, decarboxylating 1 | | Q9LSU0 | Carbohydrate Metabolism | -0.50548 |
| 74 | Proteasome subunit alpha type-3 |  | Q0JGZ6 | Translational Modification | -0.50548 |
| 75 | Fructokinase-1 |  | Q69TH8 | Carbohydrate Metabolism | -0.48449 |
| 76 | Putative 12-oxophytodienoate reductase 4 |  | Q08480 | Lipid Metabolism | -0.47767 |
| 77 | Adenylate kinase 4 |  | P30298 | Amino Acid Biosynthesis | -0.47437 |
| 78 | Sucrose synthase 2 |  | P40392 | Carbohydrate Metabolism | -0.46342 |
| 79 | Ras-related protein RIC1 |  | Q9LST6 | Signaling and Cellular Process | -0.44154 |
| 80 | Proteasome subunit beta type-2 |  | Q7FAE1 | Translational Modification | -0.43981 |
| 81 | Momilactone A synthase |  | Q7XN11 | Secondary Metabolites Biosynthesis | -0.43299 |
| 82 | Gamma-aminobutyrate transaminase 1, mitochondrial |  | P14717 | Amino Acid Biosynthesis | -0.42072 |
| 83 | Phenylalanine ammonia-lyase |  | P0C5A4 | Secondary Metabolites Biosynthesis | -0.40684 |
| 84 | Late embryogenesis abundant protein, group 3 |  | Q42971 | Stress Response | -0.39736 |
| Exclusive proteins found in 0.10% PF-68 when compared to optimum (0.04% PF-68) concentration | | | | | |
| 85 | Asparagine synthetase [glutamine-hydrolyzing] 1 |  | Q10MX3 | Amino Acid Biosynthesis | N/A |
| 86 | Chalcone--flavonone isomerase |  | Q84T92 | Secondary Metabolites Biosynthesis | N/A |
| 87 | Cinnamyl alcohol dehydrogenase 2 |  | Q6ZHS4 | Phenylpropanoid Biosynthesis | N/A |
| 88 | Glutaredoxin-C6 |  | P55142 | Transporter Protein | N/A |
| 89 | GTP-binding nuclear protein Ran-1 |  | Q7F7I7 | Transporter Protein | N/A |
| 90 | Histidinol dehydrogenase, chloroplastic | | Q5NAY4 | Amino Acid Biosynthesis | N/A |
| 91 | Histone H2B.5 |  | Q94JE1 | Signaling and Cellular Process | N/A |
| 92 | IAA-amino acid hydrolase ILR1-like 7 |  | Q8H3C9 | Protein Biosynthesis | N/A |
| 93 | Late embryogenesis abundant protein 1 |  | A3AHG5 | Stress Response | N/A |
| 94 | Nitrogen regulatory protein P-II homolog |  | Q6AUR2 | Nitrogen Metabolism | N/A |
| 95 | NPL4-like protein |  | Q9AS33 | Translational Modification | N/A |
| 96 | Probable 5'-adenylylsulfate reductase 1, chloroplastic |  | Q6Z4A7 | Stress Response | N/A |
| 97 | Probable D-2-hydroxyglutarate dehydrogenase, mitochondrial |  | Q7XI14 | N/A | N/A |
| 98 | Protein IN2-1 homolog B |  | Q8H8U5 | Amino Acid Biosynthesis | N/A |
| 99 | Ribulose-phosphate 3-epimerase, cytoplasmic isoform |  | Q9SE42 | Amino Acid Biosynthesis | N/A |
| 100 | RNA-binding protein Y14A |  | B7FAL5 | Transporter Protein | N/A |

N/A indicates not available
